# Tick ecology and host-finding efficiency interact to determine disease risk: a model of heartwater dynamics

**DOI:** 10.1017/S0031182025100553

**Published:** 2025-08

**Authors:** Adam M. Fisher, Hannah Rose Vineer

**Affiliations:** Department of Infection Biology and Microbiomes, Institute of Infection, Veterinary and Ecological Sciences, University of Liverpool, Neston, Cheshire, CH64 7TE, UK

**Keywords:** enzootic stability, heartwater, mathematical model, ℛ_0_, *Rickettsia*, tick-borne disease, vectors

## Abstract

Heartwater is a tick-borne disease (TBD) of wild and livestock ruminants that threatens food security and the economy throughout much of Africa. Furthermore, the geographic range of heartwater is expanding and is predicted to continue doing so. Despite this, our understanding of heartwater dynamics lags far behind that of many other TBDs. We are therefore limited in our ability to design effective disease control strategies. In this study, we derive and analyse a mathematical model of heartwater dynamics. We analyse our model to predict the most influential parameters for disease risk, both in terms of new outbreaks and in heartwater-endemic regions. We show that the host-finding efficiency of ticks is the most influential parameter affecting outbreak risk. Also, outbreak risk is highly sensitive to the impact of the heartwater pathogen on tick fitness – a previously unexplored concept for any TBD system. In areas where heartwater is established, we show that disease can be controlled via enzootic stability (prolonged host immunity attained via frequent pathogen exposure). However, the maintenance of enzootic stability was dependent on several ecological and physiological parameters. Regarding practical output, we suggest prioritizing tick control measures during periods when ticks are most active in terms of dispersing towards hosts, so as to mitigate heightened outbreak risk. In addition, given the specificity of conditions required for enzootic stability, we caution against relying solely on enzootic stability for long-term heartwater protection. More broadly, our study highlights important tick life history parameters that have been neglected by previous TBD models.

## Graphical abstract

**Figure:**
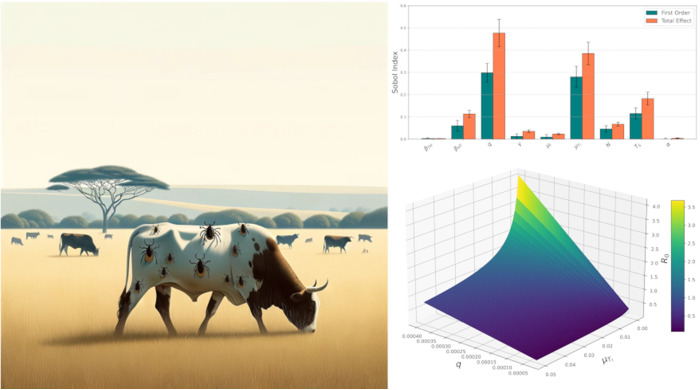

## Introduction

Heartwater is a tick-borne disease (TBD) affecting wild and livestock ruminants throughout southern Africa. The disease is transmitted via the bites of *Amblyomma* spp. ticks infected with the rickettsial bacterium *Ehrlichia ruminantium*. The clinical manifestation of heartwater can be characterized by severe neurological symptoms, vascular damage and rapid progression to death in susceptible animals (Van de Pypekamp and Prozesky, 1987). Moreover, a recent study estimated the total economic cost of heartwater to be 1.2 billion ZAR per annum (equivalent to 69 million US dollars at the time of writing) in South Africa alone, with most of these costs coming by way of increased livestock mortality (van den Heever et al., 2022). Worse still, *Amblyomma variegatum* has carried heartwater to several African islands and parts of the Caribbean, demonstrating that the range of *Amblyomma* spp. need not be limited to mainland Africa (Kelly et al., 2011). Furthermore, climate models predict that the ecological niche of *Amblyomma* spp. will broaden in the coming decades, increasing the likelihood of heartwater spreading to new regions (Wagner et al., 2002; Estrada-Peña et al., 2007). Despite the current and potential future impacts of heartwater, the heartwater literature is surprisingly scarce relative to that of other TBDs. As such, we still lack an awareness of many of the fundamental epidemiological drivers of heartwater dynamics.

Theoretical models of disease epidemiology can provide us with a mechanistic understanding of how diseases spread; this foundational knowledge can then be used to inform disease control strategies. Such models have greatly benefitted our understanding and ability to control many TBDs (Norman et al., 2016). For example, theoretical models have guided efforts to control louping ill virus (LIV), a disease spread by *Ixodes ricinus* to sheep and red grouse. Here, models identified the treatment of hosts with acaricides as a practical and effective approach for limiting LIV prevalence (Porter et al., 2011, 2013). In addition, theoretical models have shown that high deer densities can create a ‘dilution effect’ by acting as non-competent disease reservoirs; this can reduce disease risk for both tick-borne encephalitis (TBE) and Lyme disease (LD) by reducing host-to-tick transmission rates (Bolzoni et al., 2012; Dunn et al., 2013).These findings have greatly influenced the dialogue and advice surrounding how deer populations should be managed with respect to TBD (Pepper et al., 2020). Furthermore, management of babesiosis (a tick-borne protozoan disease of livestock) has been influenced by theoretical models demonstrating the efficacy of enzootic stability for controlling disease (Jonsson et al., 2012). Here models show that, in areas of high *Babesia* spp. prevalence in ticks, low rates of clinical infection in livestock can be achieved by maintaining high frequencies of host immunity via consistent exposure to infected ticks (Mahoney and Ross, 1972; Bock et al., 2004).

Despite the clarity provided by the aforementioned theoretical models, their findings are not directly relevant to heartwater due to important epidemiological nuances associated with the heartwater disease system. Firstly, host resistance to heartwater can arise via at least three routes: (1) recovery from infection, (2) maternally derived antibodies and (3) innate immunity in the early stages of life (Norval et al., 1995). Moreover, immunity to heartwater can be maintained via repeated exposure to infected ticks (Deem et al., 1996a). While these patterns of immunity do share some crossover with the previously mentioned disease systems (e.g., juvenile livestock can inherit immunity to LIV and babesiosis), none of these previous studies model infection dynamics in a way that is entirely consistent with the heartwater system. For example, many models of babesiosis account for transovarial transmission of *Babesia* spp. in ticks (Saad-Roy et al., 2015; Wang et al., 2020), a phenomenon not known to occur with *E. ruminantium* and *Amblyomma* spp. ticks. Secondly, LIV, TBE, and LD are all spread via *Ixodes* spp. ticks, which have substantially different life histories to *Amblyomma* spp. (the tick genus responsible for spreading heartwater). Most notably, *Amblyomma* spp. actively move towards hosts (Godfrey et al., 2011; Marshall et al., 2025), whereas *Ixodes* spp. adopt a sit-and-wait strategy (Van Es et al., 1999). As such, studies modelling diseases spread by *Ixodes* spp. often utilize a simple approach to formulating infection rates, whereby host-tick contact rates are scaled only by the frequency of hosts and ticks, but variation in the innate host-finding capabilities of ticks is ignored.

Although the theoretical literature on heartwater is scarce, there are at least three studies that have used mechanistic models to predict heartwater dynamics. O’Callaghan et al. (1998) developed a compartmental heartwater disease model in which *Ehrlichia ruminantium* dynamics, host demographics, and tick density were modelled explicitly. This study provided many valuable insights, particularly regarding how tick attachment rate could modulate infection dynamics. Specifically, the study highlights how enzootic stability is positively associated with innate and maternally derived immunity, and maintained by high tick attachment rates. A second study by the same group builds upon these findings by demonstrating how vaccination regimes can be used to transition herds to a state of enzootic stability while minimizing deaths (O’Callaghan et al., 1999). Despite providing valuable insights, these studies do not conduct a comprehensive analysis of the model parameters and their interactive effects. Moreover, these models do not explicitly analyse the basic reproductive number (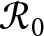) – the number of new infections generated per infected individual – and are therefore limited in the extent to which they can predict new outbreaks. Finally, Yonow et al. (1998) re-parameterized a host–mosquito model to predict heartwater prevalence in livestock. The authors concluded that heartwater always reaches a high prevalence where it is found; thus, enzootic stability, not disease prevention, is the only way to manage infections. However, this study did not model host immunity, and is therefore likely to overestimate the rate of disease spread and disease prevalence. As such, there are still significant gaps in our fundamental understanding of the drivers of heartwater dynamics.

In this study, we address the aforementioned knowledge gaps by building and analysing a model of heartwater dynamics. As theoretical models become more complex, analysis and interpretation can become more challenging, diminishing the extent to which the model can improve our mechanistic understanding of the system (Keeling and Rohani, 2008). Our approach was, therefore, to prioritize model interpretability by optimizing the trade-off between model simplicity and biological realism. This permits a global analysis of the model parameters, allowing for clear mathematical delineations between cause-and-effect. We use our model to predict how the parameters, and their interactions, impact heartwater disease risk, both in terms of new outbreaks and long-term prevalence.

## Methods

To investigate the mechanisms of heartwater dynamics, we derive and analyse a set of differential equations that model host–tick interactions and pathogen transmission. The data available for model parameterization are largely limited to *Amblyomma hebraeum* and cattle (*Bos taurus* and *Bos indicus*). Thus, our model output is most relevant to understanding disease dynamics in livestock systems involving the aforementioned species. The model is parameterised such that one time step represents 24 h.

### The model – host dynamics

We employ an SIR framework in which hosts fall into one of the following classes: susceptible (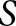) – not infected nor immune, infected (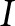) – currently infected or recovered (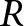) – currently immune. Hosts are born at rate 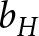 which is multiplied by the density-dependent term 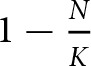, where 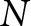 is the total host population density (
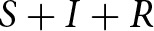
) and 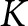 is the maximum density of cattle per km^2^. As a result, population growth is asymptotic. Considerable evidence suggests there is vertical transmission of immunity from mothers to calves (Norval et al., 1995; Deem et al., 1996b). As such, we assume that all offspring of recovered hosts are born into the recovered class. Some evidence suggests newborn calves possess age-related immunity despite being born of non-immune mothers (Du Plessis and Malan, 1987); hence, the proportion of offspring of susceptible hosts born into the recovered category is 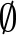. We assume that hosts do not reproduce when they have an active infection.

The frequency of tick bites is proportional to the total density of ticks (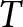) and tick host-finding rate (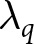). Due to the data available for parametrization (see below), and because we want to directly model variation in innate host-finding traits, we derive 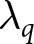 from the per-tick probability of finding a host in 24 h. We define parameter 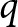 as the probability a tick will find a host in 24 h when total host density 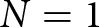, 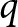 is thus a measure of innate ‘host-finding efficiency,’ and for concision, we refer to it as such from here on. Therefore, the per-tick probability of finding a host in 24 h is 
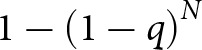
. We then log transform 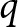 and scale by 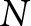 to get the following daily host-finding rate:
(1)
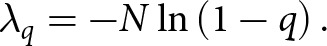


This conversion is standard practice when dealing with time-specific discrete probabilities in continuous time models; it ensures that the correct cumulative host-finding rate is maintained when the model is integrated over time steps smaller than 1. The frequency at which a specific class of hosts is bitten by an infected tick is proportional to infected tick density (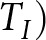, and inversely proportional to the density of said host class relative to total host density. Thus, the overall rate at which 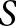 are bitten by 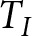 is 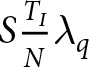. Once bitten by an infected tick, the probability a host becomes infected is determined by the per-bite tick-to-host transmission probability 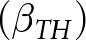. Infected hosts recover at rate 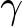 and suffer an excess per capita mortality rate of 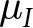. Given this paper’s focus on outbreak potential and short-term disease dynamics in long-lived (when healthy) hosts, we assume only infected hosts die.

After recovering from infection, hosts enter the recovered class. Immunity in recovered hosts wanes at rate 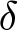. Immunity can be prolonged through repeated bites from infected ticks that occur prior to immunity waning; this is a well-known phenomenon based on field data (Norval et al., 1995). However, the precise relationship between infecting tick bite frequency and the extension of the immune period is not known. Thus, we simply scale the overall rate at which recovered individuals become susceptible by the rate at which recovered hosts receive infecting bites. As such, the full equations describing host population and infection dynamics are:

(2)
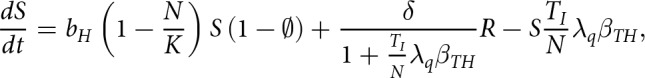

(3)
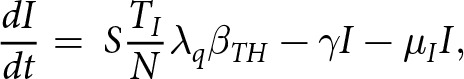

(4)
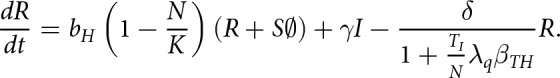

### The model – tick dynamics

For simplicity, we ignore tick population age structure. As such, the total tick population 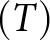 falls into two classes: susceptible (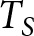) and infected (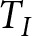). Ticks are born at rate 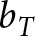. Like in the host population, tick population birth rate is density-dependent, and the strength of this density dependence is determined by the size of the host population (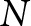) and the maximum number of ticks each host can support (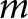). Thus, ticks reproduce at rate 
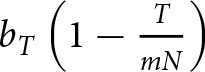
, and all ticks are born susceptible. Susceptible ticks become infected at a rate proportional to host finding rate (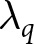), the relative frequency of infected hosts 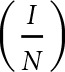, and the per-bite host-to-tick transmission probability 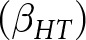. Ticks starve to death at a rate inversely proportional to the length of time in days a tick can survive without feeding (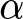); this starvation rate is then scaled by host finding rate (
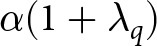
). Infected ticks can suffer excess mortality at rate 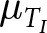. We assume that infected ticks do not recover after becoming infected. As such, the full equations describing tick population and infection dynamics are:

(5)
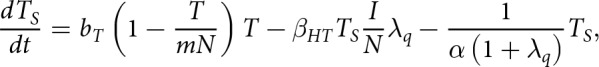

(6)
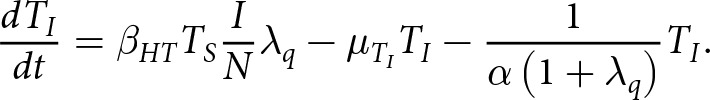

### Parameterization

The number of studies used to inform parameter values was limited due to the scarcity of work that has directly measured fundamental epidemiological attributes of *Ehrlichia ruminantium*. When determining the parameter ranges to be used in the model analysis, we partially relied on the data ranges observed in the aforementioned studies. However, given the inherently limited sample sizes of most empirical field-based studies, these ranges almost certainly do not capture the true range. As such, parameter ranges were extended in accordance with our knowledge and expert judgement. For parameter values not taken directly from the primary literature, parameterization decisions are justified below.

Values for 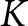 were informed by the cattle density map provided by the Food and Agriculture Organization of the United Nations (UN, 2015). Specifically, we observed the typical range of cattle densities in Botswana and Zimbabwe, as both countries are heavily affected by heartwater (van den Heever et al., 2022; Ramotadima et al., 2023).

The host-finding rate of wild ticks is inherently difficult to measure directly due to the low feasibility of tracking individual ticks. However, the daily tick attachment rates in farmed cattle in Zimbabwe have been quantified (O’Callaghan et al., 1998). Here, ticks were removed from cattle before returning 24 h later to count recently attached ticks. The authors reported a maximum daily attachment rate of 0.5 ticks per cow. In our model, the discrete time daily tick attachment probability per host is defined as 
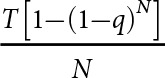
; thus, if we assume that the author’s observations were taken when ticks and hosts were at carrying capacity and set tick attachment rate to 0.5, we get the following:
(7)
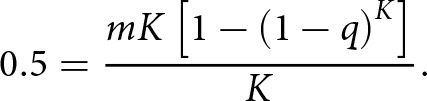


This expression can now be rearranged and simplified to give the corresponding formula for 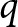,
(8)
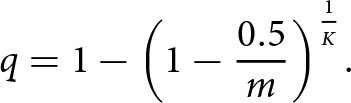


Assuming host and tick density were at equilibrium at the time of the aforementioned field study, we can use this formula to give estimates for 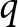 across a range of ecological scenarios. We can calculate the minimum value of 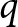 by setting 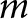 and 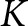 to their maximum values (100 and 200, respectively) – giving 0.00003. Likewise, to generate the maximum value of 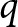, we set 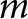 and 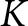 to their minimum values (25 and 50, respectively) – giving 0.0004. Given that 0.5 is likely to underestimate the maximum tick attachment rate due to observational error (i.e., failing to count all ticks on a cow), the default value of 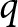 is set to its maximum value of 0.0004 (Table 1).

**Table 1.** Model parameters

Symbol	Definition	Default value	Range	Reference
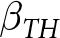	Per-bite transmission probability (tick-to-host)	0.8	0.7−0.9	(Norval et al., 1990)
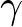	Recovery rate of infected hosts	0.05	0.01– 0.1	(Camus et al., 1996)
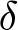	Rate of immunity loss in recovered hosts and those with inherited immunity	0.005	–	(Stewart, 1987)
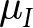	Infected host death rate	0.067	–	(Van de Pypekamp and Prozesky, 1987)
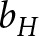	Host birth rate	0.02	–	–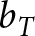	Tick birth rate	0.08	–	–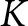	Host carrying capacity (hosts per km^2^)	100	50−200	(UN, 2015)
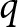	Tick host-finding efficiency – the probability a specific tick will find a host over 24 h when 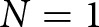	0.0004	0.00003−0.0004	(O’Callaghan et al., 1998)
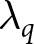	Per-tick host-finding rate derived from 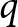 and 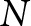	–	–	–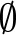	The probability a susceptible dam births an immune offspring	0.5	0–1	(Du Plessis and Malan, 1987; Norval et al., 1995)
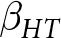	Per-bite transmission probability (host-to-tick)	0.12	0.05−0.32	(Peter, 1995)
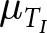	Increase in tick death rate caused due to *Ehrlichia ruminantium* infection	0	0−0.05	–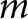	Maximum number of ticks a single host can sustain	50	25−100	(Heylen et al., 2023)
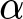	The average number of days a tick can survive without feeding	518	300−600	(Fielden and Rechav, 1996)

Default values correspond to the value of the parameter when said parameter is not being subject to sensitivity analysis; a lack of a parameter range indicates that parameter was fixed throughout the study. The model is parameterized such that each time step is 24 h; thus, all rates have units day^−1^.

Regarding parameter 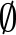, there is some evidence that newborn calves have innate immunity to *E. ruminantium*, regardless of their dam’s immune status (Du Plessis and Malan, 1987). However, current data are too scarce to confidently ascertain the frequency at which this innate immunity occurs. Moreover, another study found that *E. ruminantium* immunity in calves was likely due to the presence of maternally derived antibodies (Norval et al., 1995). Given the fact that heartwater studies tend to focus on areas where disease is already endemic, it may be that the apparent ‘innate’ immunity observed in previous studies was simply the product of immunity in the adult population leading to widespread maternally derived immunity. Given this uncertainty, we set 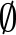 to an ambiguous default value of 0.5 and allow 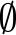 to vary across its entire range (0–1) during analysis.

The current study is not concerned with analysing the impact of intrinsic birth rates on heartwater epidemiology; thus, 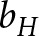 and 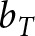 were given arbitrary values of >0; this simply assumes that, in the absence of the processes being modelled, host and tick populations persist through time. Moreover, we set 
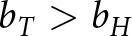
 to reflect the inherently larger brood sizes and shorter generation times of invertebrates (ticks) relative to vertebrates (cattle).

Finally, although there is no evidence that *Ehrlichia ruminantium* reduces longevity in *Amblyomma* ticks, this has not been explicitly tested to our knowledge. Indeed, other rickettsial bacteria can increase mortality in other tick species. For example, *Rickettsia ricketsii*, the bacterium responsible for Rocky Mountain spotted fever, significantly increases mortality in its tick vector *Dermacentor andersoni* (Azad and Beard, 1998; Niebylski et al., 1999). In addition, *Rickettsia amblyommatis* infection was recently shown to have negative fitness consequences for *Amblyomma americanum* (Richardson et al., 2022). As such, the potential for *E. ruminantium* infection to increase tick death rate (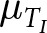) is included in the model as an exploratory parameter. Given that ticks carrying *E. ruminantium* are known to be competent vectors of heartwater, we assume that additional deaths due to *E. ruminantium* in *Amblyomma* would, if present, be relatively low. Hence, 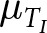 is given a conservatively low and narrow parameter range and is assumed to have a default value of 0 (Table 1).

### Analysis – disease-free equilibrium

To determine the stability of the disease-free equilibrium (i.e., whether or not heartwater could spread in a naïve population), we calculated the basic reproductive number (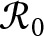) using the Next-Generation Matrix method (Diekmann et al., 2010). A detailed mathematical derivation of 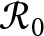 can be found in the supplementary material.

After calculating 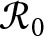, we used Latin Hypercube Sampling via the scipy.stats.pmc module in Python’s SciPy library (Virtanen et al., 2020) to perform a global sensitivity analysis of 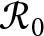; this allowed for a qualitative visualization of the effect of each parameter on 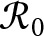. Then, using the sobol.analyze function from Python’s SALib.analyze module (Herman and Usher, 2017), we used Sobol’s Sensitivity Index to formally quantify the first and total order effect of each 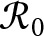 parameter. First-order effects quantify the isolated impact of variation in a parameter, whereas the total order effect is the sum of the first-order effect and the impact of the focal parameter via interactions with other parameters. We then observed the parameters that had the largest disparity between their first and total order effects (indicating the presence of important interactions), and visualized interactions between these parameters using 3D surface plots. Parameter ranges were defined as in Table 1.

### Analysis – equilibria under established disease

Given the dependence of tick abundance and activity on environmental conditions (Gaff et al., 2020; Hancock et al., 2011), models without seasonal effects (as is the case with this model) are of limited use when attempting to accurately predict the long-term dynamics of TBDs. Nevertheless, our model can be used to understand the general impact of model parameters on heartwater dynamics through time. As such, we graphically analysed the equilibria of the host and tick classes once the disease had established. To do this, we solved our model by integrating our differential equations with a step size of 0.1 using the Livermore Solver for ordinary differential equations method implemented in Python’s SciPy (scipy.integrate.odeint) library (Virtanen et al., 2020). Given that our model is inherently asymptotic due to density-dependent population growth, we assumed that population frequencies at 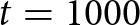 approximated to the true equilibrium frequencies. Thus, for each run of 1000 time steps, we changed the value of a focal parameter and then plotted variation in the relative density of host classes at 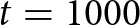 against variation in the focal parameter. From here, we could observe how our model parameters affected the equilibrium frequencies of infected, susceptible and immune individuals. Given the possibility for the establishment of enzootic stability in heartwater systems (O’Callaghan et al., 1998), we were particularly interested in the parameter space under which 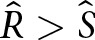. Parameter ranges were defined as in Table 1.

All analyses were conducted using Python 3.10 in a Jupyter Notebook environment (Kluyver et al., 2016). Fully annotated model and analysis code can be found in the supplementary material.

## Results

Because all 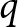 values are ≤0.0004 (see Table 1), the approximation 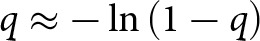 is extremely tight (e.g., 0.0004 vs 0.00040008 at the upper bound, a 0.02 % difference). Therefore, for brevity, we report results directly in terms of 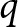.

After calculating 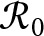 (see supplementary material) and rearranging the expression for 
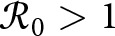
, we determined that the disease-free equilibrium is unstable when

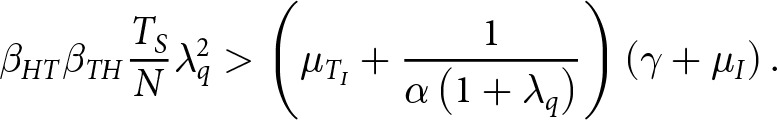


**Figure 1.**
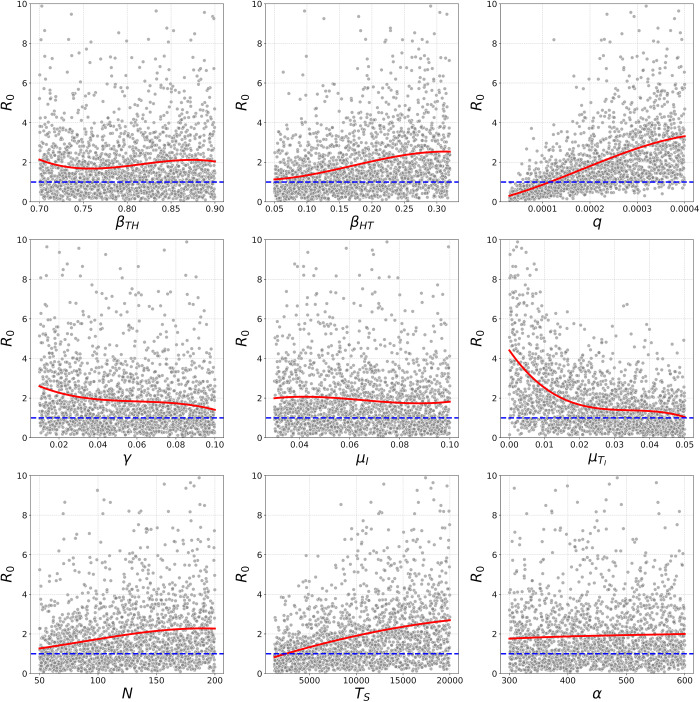
Variation in 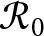 in response to Latin Hypercube Sampling of all the 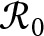 parameters. Solid red lines indicate fitted third-order polynomial regression models, and dashed blue lines indicate the threshold 
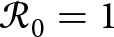
. Parameter definitions and sampling ranges are as defined in Table 1.

Our global sensitivity analysis of 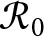 using Latin Hypercube Sampling revealed that tick host-finding efficiency (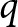), per-bite host-to-tick transmission probability (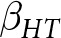), host density (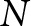) and the density of susceptible ticks (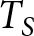) all had a qualitatively positive association with 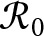 (Fig. 1). Moreover, the rate of additional tick mortality caused by *Ehrlichia ruminantium* infection (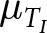) had a qualitatively negative and non-linear association with 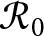 (Fig. 1). Among all parameters, the stability of the disease-free equilibrium seemed most sensitive to 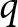, as 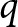 was the only parameter that could drive 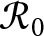 substantially below 1 (Fig. 1).

**Figure 2.**
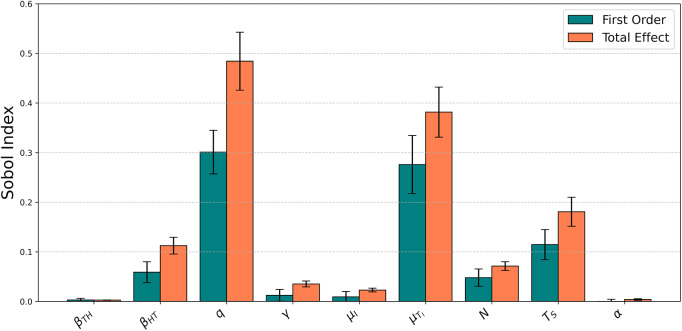
The sensitivity of 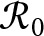 (quantified using Sobol’s Sensitivity Index) in response to variation in each of the 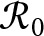 parameters. First-order effects (left bars) indicate the sensitivity of 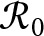 to isolated variation of a given parameter, total effects (right bars) indicate the summed sensitivity of 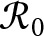 to isolated effects and interactive effects involving the focal parameter. Error bars indicate 95% confidence intervals calculated using bootstrapped Monte Carlo simulations.

Quantitative analysis of the 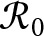 parameters using Sobol’s Sensitivity Index showed that 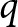 and 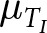 were by far the parameters most influential for determining 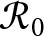 (Fig. 2). In addition, there was a sizeable difference between the first order and total effects of both 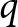 and 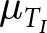, indicating the presence of important interactions involving 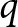 and 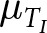. There were also considerable total effects, and sizeable differences between the first order and total effects (once again, indicating the presence of interactions) for 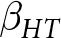, 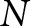, and 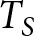 (Fig. 2). To this end, our quantitative analysis using Sobol’s Index corroborates the main findings of our qualitative Latin Hypercube analysis in highlighting 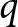, 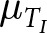, 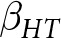, 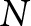, and 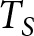 as the parameters most influential for determining 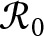.

**Figure 3.**
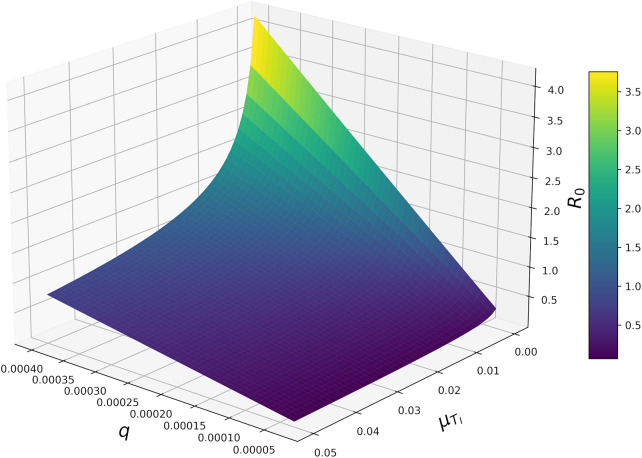
Host-finding efficiency 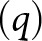 and additional tick mortality rate due to infection 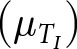 modulate 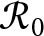 via an interactive effect of considerable magnitude.

There was an interaction of considerable magnitude between 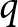 and 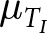 (Fig. 3). Specifically, when 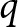 was at its maximum value (0.0004), 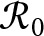 increased exponentially as 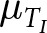 decreased; the magnitude of this trend was drastically reduced as 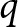 decreased to its minimum value (0.00003). Furthermore, the strong positive relationship between 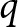 and 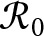 (which is most obvious when 
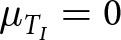
) was dampened as 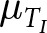 increased (Fig. 3). In other words, increasing 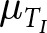 reduced the extent to which 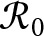 could be increased by increasing 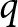. As a result, stable disease-free equilibrium (
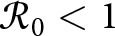
) can be reached when 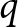 is low, or when the there is an increase in tick mortality due to *E. ruminantium* infection (Fig. 3). There were also interactive effects between 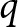 and 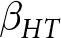, 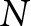, and 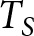, such that the positive impact of 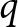 on 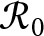 was dampened in response to reductions in 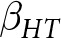, 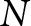, or 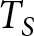. However, these interactions were relatively weak, and the attainment of a stable disease-free equilibrium was predominantly determined by variation in 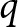 (Supplementary material; Figure S1).

**Figure 4.**
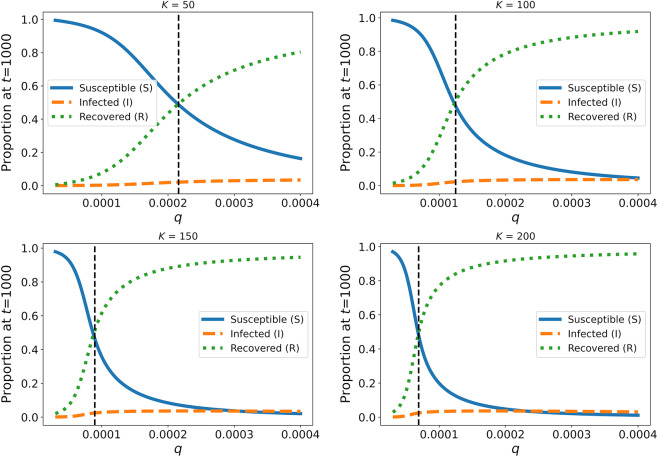
The impact of tick host-finding efficiency 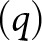 and host carrying capacity 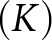 on the frequency of susceptible 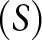, infected 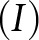 and recovered/immune 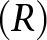 hosts at 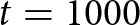 (presumed equilibrium frequency). The vertical dashed line represents the *q* threshold – the point at which the frequency of 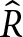 becomes greater than that of 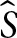.

By analysing the equilibrium frequency of susceptible (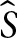), infected (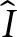) and recovered/immune (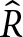) hosts, we show that the relative frequency of 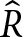 increases with increasing tick host-finding efficiency (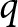) (Fig. 4). The relative frequencies of 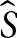, 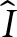, and 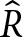 for a given value of 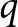 were modulated by host carrying capacity (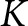) such that, as 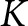 increased, the frequency of 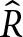 relative to 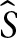 and 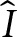 increased. Furthermore, increasing 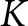 caused the threshold at which the frequency of immune individuals exceeds the frequency of susceptible individuals (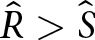, referred to hereon as the ‘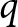 threshold’) to occur at lower values of 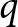 (Fig. 4). The relative equilibrium frequency of infected individuals remains low (<4%) across the parameter space (Fig. 4). Variation in the rate at which susceptible hosts produce recovered/immune offspring had very weak/negligible negative impact on the 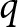 threshold (Supplementary material; Figure S2).

**Figure 5.**
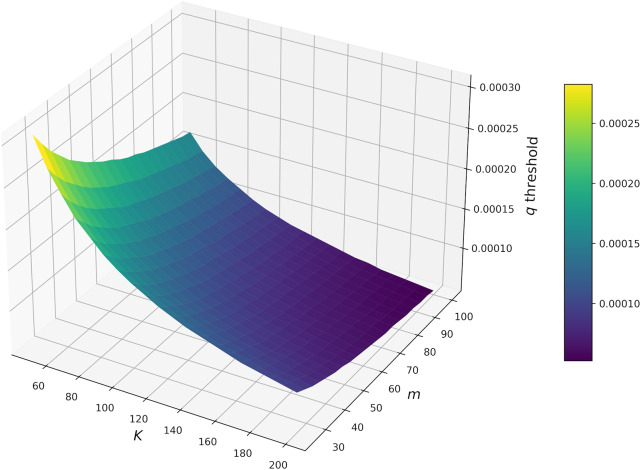
Host-carrying capacity 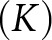 and the maximum number of ticks a single host can sustain 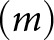 interact to determine the 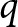 threshold (i.e., the value of 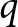 needed to drive the equilibrium frequency of recovered/immune hosts to become greater than that of susceptible hosts).

Finally, we observed an interactive effect of host carrying capacity (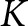) and the maximum number of ticks a single host can sustain (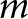) on the 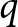 threshold (Fig. 5). Here, the 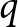 threshold was highly sensitive to variation in 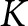, such that increasing 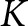 greatly reduced the 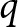 threshold; this effect was dampened as 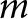 increased (Fig. 5).

## Discussion

For any disease, a mechanistic understanding of epidemiology is essential for designing effective control strategies. In this study, we derived and performed a global analysis on a mechanistic model of heartwater dynamics. In doing so, we fill many of the knowledge gaps remaining after previous work. Specifically, we show that 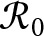 is sensitive to several parameters (Figs 1 and 2); however, innate host-finding efficiency (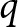) was the most influential parameter in determining whether or not heartwater would spread through a naïve population. Furthermore, we show that the presence of a negative fitness consequence for ticks of carrying the heartwater-causing pathogen (*Ehrlichia ruminantium*) greatly dampens the impact of 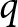 on 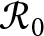 to maintain a stable disease-free equilibrium (Fig. 3). In addition, we found that the equilibrium frequencies of host compartments (susceptible, infected, or recovered/immune) after disease had established, were heavily modulated by interactions between tick host-finding efficiency (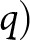, host carrying capacity (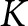) and the maximum number of ticks per host (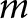) (Figs 4 and 5). The current proposed control strategies for heartwater largely fall into two distinct categories: (1) preventing disease by protecting livestock from exposure to infected ticks and (2) controlling disease via prolonged population immunity – enzootic stability (Stachurski et al., 2019). Below, we discuss the relevance of our results to both of these disease control strategies, and to TBD models more generally.

### Predicting and preventing outbreaks


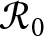
 – the number of new infections created per infected individual per time step – is one of the most commonly used metrics of outbreak risk and rate of disease spread. Previous TBD studies often cite tick and host density as the primary determinants of 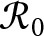 (Rosà and Pugliese, 2007; Harrison et al., 2011). We found that, in terms of impact on 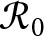, variation in tick host-finding efficiency (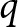) was far more important than variation in host (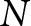) or tick density (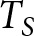) (Fig. 2). Given our model design, this finding is somewhat unsurprising, as 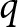 affects the rate at which hosts *and* ticks become infected, 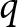 therefore becomes a squared term in the 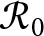 expression (see supplementary material). Nevertheless, this finding does highlight tick host-finding efficiency as an important but underappreciated determinant of TBD outbreak risk. In more general terms, this result highlights the importance of understanding variation in tick behaviour for predicting disease spread.

Heartwater outbreaks can be particularly damaging to naïve populations with no prior immunity (Van de Pypekamp and Prozesky, 1987; van den Heever et al., 2022). Moreover, new outbreaks expand the geographical range of heartwater. As such, precautions should be taken to minimize exposure risk in heartwater-naïve livestock populations. Based on our results, strategies employed to protect livestock from heartwater exposure should be prioritized during periods of high host-finding efficiency in *Amblyomma* spp. ticks. For example, if the use of acaricide to control tick populations is limited due to environmental or financial constraints, acaricide treatment should be prioritized during periods of high tick activity. In addition, human behaviours that increase the risk of heartwater introduction to new areas (e.g., importing livestock from heartwater endemic areas) should be limited as much as possible to periods of low tick activity. However, in order to guide these measures as accurately as possible, empirical data on how host-finding efficiency in *Amblyomma* spp. varies across environmental gradients are needed.

Although studies measuring the impact of environmental variables on activity rates in *Amblyomma* spp. are extremely scarce, there have been several such studies conducted on *Ixodes* spp. Here, it has been shown that environmental variables such as temperature, humidity and altitude can have significant effects on questing (sit-and-wait host-finding) behaviour (Vail and Smith, 1998; Gern et al., 2008). Despite this, TBD models often assume a constant tick activity rate (Yonow et al., 1998). Based on our findings, assuming constant tick activity is likely to lead to erroneous predictions regarding the disease risk posed by a given tick population. Though admittedly, due to greater scope for behavioural variation, assuming constant tick activity is likely to be a much greater issue for models predicting disease spread in ticks that actively seek hosts (e.g., *Amblyomma* spp.), as opposed to questing ticks (e.g., *Ixodes* spp.).

Empirical studies have observed a sizeable impact of rickettsial infection on tick longevity (Azad and Beard, 1998). For example, Niebylski et al. (1999) showed that infection with *Rickettsia ricketsii* (the pathogen responsible for Rocky Mountain spotted fever) increases mortality rates in *Dermacentor andersoni* by 3.3 times relative to control/uninfected individuals. Niebylski et al. (1999) also showed that this impact on mortality increases with temperature. Despite these findings, we are, to the best of our knowledge, the first to build a mechanistic model that considers the potential impact of TBD pathogens on tick fitness. We found that increased tick death due to *Ehrlichia ruminantium* infection (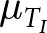) had a strong negative effect on 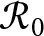 (Figs 1 and 2). We also found that the impact of host-finding efficiency (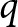) on 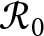 was heavily modulated by 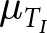; such that even a small increase in 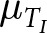 could drastically reduce 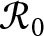 when 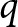 was high (Fig. 3). If the tendency for pathogens of TBDs to negatively impact the fitness of their tick vectors is widespread, then our result highlights the importance of accounting for these fitness effects in TBD models. Moreover, if the impact of TBD pathogens on tick fitness interacts with environmental factors, as was shown by Niebylski et al. (1999), then this additional layer of biological nuance that should be accounted for in future TBD models; this is particularly true for studies that aim to model seasonality.

### Managing enzootic stability

States of enzootic stability are characterized by a high frequency of prolonged resistance to clinical disease in host populations. In TBDs, this immunity is a direct consequence of persistent challenge by infected ticks. As a result, enzootic stability can provide protection from clinical disease without needing to intensively control tick populations (Jonsson et al., 2012). Both theoretical models and field studies have demonstrated the effectiveness of enzootic stability for controlling babesiosis (a TBD caused by protozoa) in cattle (Smith, 1983; Regassa et al., 2003). Our model shows that the frequency of immunity scales with tick challenge to keep infection rates low at equilibrium (Fig. 4). Thus, in theory, our results show that enzootic stability can be achieved for heartwater. This finding supports the results of an earlier theoretical heartwater study (O’Callaghan et al., 1998). However, model-predicted infection rates under equilibria do not take into account infection rates from early in an outbreak, when host immunity may lag behind infected tick challenge. Because of this, caution must be taken when attempting to use our equilibrium-analysis to predict the actual death toll of a heartwater outbreak. Nevertheless, our model can be used to generate predictions about the conditions under which high levels of immunity are established, which may protect host populations from future spikes in tick abundance and/or activity. We show that the frequency of immunity is highly sensitive to tick host-finding efficiency (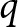) and cattle density, with the highest levels of immunity occurring under high host densities and high 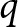 (Fig. 4). Moreover, this relationship is modulated by the abundance of ticks per host (Fig. 5). Overall, our results suggest that, although enzootic stability is possible for heartwater, the frequency of immunity is sensitive to multiple factors. Thus, enzootic stability may be temporally precarious for heartwater.

Despite theoretical work demonstrating the efficacy of enzootic stability as a strategy for heartwater control (O’Callaghan et al., 1998; Yonow et al., 1998), field studies have failed to confirm these findings. It is self-evident that, in order for enzootic stability to provide adequate protection, the time interval per host between infectious tick bites must be shorter than the length of time hosts remain immune post-infection. In the case of heartwater, there are several reasons why this condition may not be met. First, the length of the immune window is known to vary greatly depending on pathogen strain and host genotype (Norval et al., 1995; Deem et al., 1996a). Second, given the highly seasonal nature of ticks, tick challenge is unlikely to be consistent throughout the year; this may lead to waning immunity during specific seasons (Jonsson et al., 2012). Several models, including this one, assume a constant immune response across hosts and a lack of seasonality; this may explain the misalignment between model predictions and field data regarding enzootic stability. Nevertheless, some evidence suggests that, by using vaccines to augment immunity, enzootic stability can be achieved (O’Callaghan et al., 1999); though vaccine efficacy must first be improved to deal with multiple *E. ruminantium* strains (Mahan et al., 1998; Stachurski et al., 2019). Based on our results, we propose that livestock vaccination should coincide with periods of low tick host-finding efficiency to compensate for waning immunity due to reductions in tick challenge; this is particularly true for low density herds (Figs 4 and 5).

### Modelling challenges

A significant challenge for future models will be accounting for inter-specific variation in host immunity and mortality, and how this interacts with different strains of *E. ruminantium*. Indeed, even across phylogenetically similar cattle species (*Bos taurus* and *Bos indicus*), the immune response to *E. ruminantium* is markedly different (Stewart, 1987). In addition, heartwater is not restricted to livestock; thus, model realism could be further improved by acknowledging wild host populations. Although our model predicts that host density has a relatively small influence on 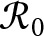 (Fig. 2), this may change in response to seasonality, particularly if peaks in wild host density coincide with peaks in tick host-finding efficiency. Models incorporating seasonal variation in tick host-finding efficiency, host-specific immune responses, and the presence of wildlife hosts, could help generate more precise directions for heartwater management strategies.

## Conclusions

Our mechanistic understanding of heartwater dynamics is lagging far behind that of many other TBDs. In this study, we attempted to address this knowledge gap. In doing so, we expose tick host-finding efficiency, and pathogen impacts on tick fitness as key parameters in determining heartwater outbreak risk in naïve populations. We also show that enzootic stability is most likely to be reached in dense livestock populations in which ticks are highly efficient at finding hosts. These findings have implications for our approach to protecting populations from disease, both in heartwater-free regions, and in areas where heartwater is already established. However, in order for the impact of our model to be fully realized, additional empirical data are needed. Specifically, data are needed to confirm how host-finding efficiency in *Amblyomma* spp. varies across seasons and environmental gradients; this will allow us to make spatio-temporal predictions of outbreak risk. Also, empirical data on the impacts of *E. ruminantium* on *Amblyomma* spp. fitness are essential for accurately predicting 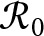. More generally, there is a desperate need for time series data of *Amblyomma* spp., both in terms of their abundance and heartwater prevalence; these data will be essential for validating predictive heartwater models. Finally, TBD models often do not account for variation in active host-finding behaviour nor the impacts of TBD pathogens on tick fitness; our results add to the growing understanding of TBD dynamics by highlighting these factors as fundamental determinants of TBD outbreak risk.

## Supporting information

Fisher and Vineer supplementary material 1 Fisher and Vineer supplementary material

Fisher and Vineer supplementary material 2 Fisher and Vineer supplementary material

## Supplementary material

The supplementary material for this article can be found at https://doi.org/10.1017/S0031182025100553.
