# Supplementary material for: Tick ecology and host-finding efficiency interact to determine disease risk: a model of heartwater dynamics
Source: Parasitology. 2025 Jul 14;152(9):975–85. doi: 10.1017/S0031182025100553 (PMC12644940; doi:10.1017/S0031182025100553)
Supplement: Fisher and Vineer supplementary material 2 — Fisher and Vineer supplementary material [file S0031182025100553sup002.docx]

**Supplementary material**

*Calculating* $\mathcal{R}_{0}$ *using the next generation matrix*

$\mathcal{R}_{0}$ represents the number of new infections that arise per infected individual. We can use $\mathcal{R}_{0}$ to determine the speed of disease spread, and the stability of the disease-free equilibrium – i.e., whether an infection can spread through a naïve population. When calculating $\mathcal{R}_{0}$, we assume the host population is absent of infections and immunity $\left( S=N \right)$; this also assumes that there have been no recent births as innate immunity is also absent. We then write the expressions for the rate at individuals enter the infected class ($F$), both in terms of hosts ($F_{I}$) and ticks ($F_{T_{I}}$):

$$F_{I}=\beta_{TH}S\frac{T_{I}}{N}\lambda_{q},$$

$$F_{T_{I}}=\beta_{HT}T_{S}\frac{I}{N}\lambda_{q},$$

and expressions for the rate at which infected individuals leave the infected class ($V$), also for hosts ($V_{I}$), and ticks ($V_{T_{I}}$):

$$V_{I}=\left( \gamma+\mu_{I} \right)I,$$

$$V_{T_{I}}=\left( \mu_{T_{I}}+\frac{1}{\alpha(1+\alpha)} \right)T_{I}.$$

We then create a matrix of the partial derivatives (the rate at which the flow of individuals in and out of the infected classes changes with respect to the frequency of infecteds). First, we create the matrix for the partial derivatives of $F$:

$$F=\left[ \begin{matrix} \frac{\partial F_{I}}{\partial I}=0 & \frac{\partial F_{I}}{\partial T_{I}}=\beta_{TH}\lambda_{q} \\ \frac{\partial F_{T_{I}}}{\partial I}=\beta_{HT}\frac{T_{S}}{N}\lambda_{q} & \frac{\partial F_{T_{I}}}{\partial T_{I}}=0 \end{matrix} \right].$$

Then, we create the matrix for the partial derivatives of $V$:

$$V=\left[ \begin{matrix} \frac{\partial V_{I}}{\partial I}=\gamma+\mu_{I} & \frac{\partial V_{I}}{\partial T_{I}}=0 \\ \frac{\partial V_{T_{I}}}{\partial I}=0 & \frac{\partial V_{T_{I}}}{\partial T_{I}}=\mu_{T_{I}}+\frac{1}{\alpha(1+\alpha)} \end{matrix} \right].$$

We can then generate the next generation matrix by multiplying $F$ by the inverse of $V$ $\left( \frac{1}{V} \right)$:

$$FV^{-1}=\left[ \begin{matrix} 0 & \frac{\beta_{TH}\lambda_{q}}{\gamma+\mu_{I}} \\ \frac{\beta_{HT}\frac{T_{S}}{N}\lambda_{q}}{\mu_{T_{I}}+\frac{1}{\alpha(1+\alpha)}} & 0 \end{matrix} \right].$$

The expression for $\mathcal{R}_{0}$ is the largest eigenvalue (aka, the spectral radius) of the next generation matrix, which, for our off-diagonal 2 x 2 matrix is:

$$\mathcal{R}_{0}=\sqrt{\frac{\beta_{HT}\beta_{TH}\frac{T_{S}}{N}\lambda_{q}^{2}}{\left( \mu_{T_{I}}+\frac{1}{\alpha(1+\alpha)} \right)\left( \gamma+\mu_{I} \right)}.}$$

Given that the disease-free equilibrium is unstable when $\mathcal{R}_{0}>1$, we can set $\mathcal{R}_{0}$ to greater than 1 and rearrange the expression to give conditions for stable/unstable disease-free equilibrium (see the Results section for this condition).

*Supplementary figures*


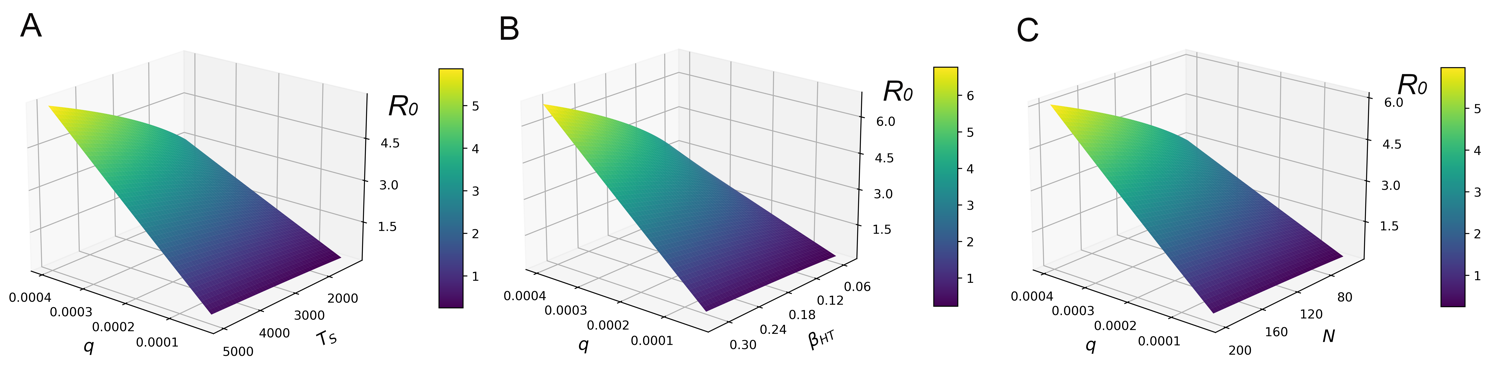


**Figure S1** – $\mathcal{R}_{0}$ is modulated by interactions between tick host-finding efficiency ($q$) and: A) susceptible tick density ($T_{S}$), B) host-to-tick transmission rate ($\beta_{HT}$), and C) host density ($N$).


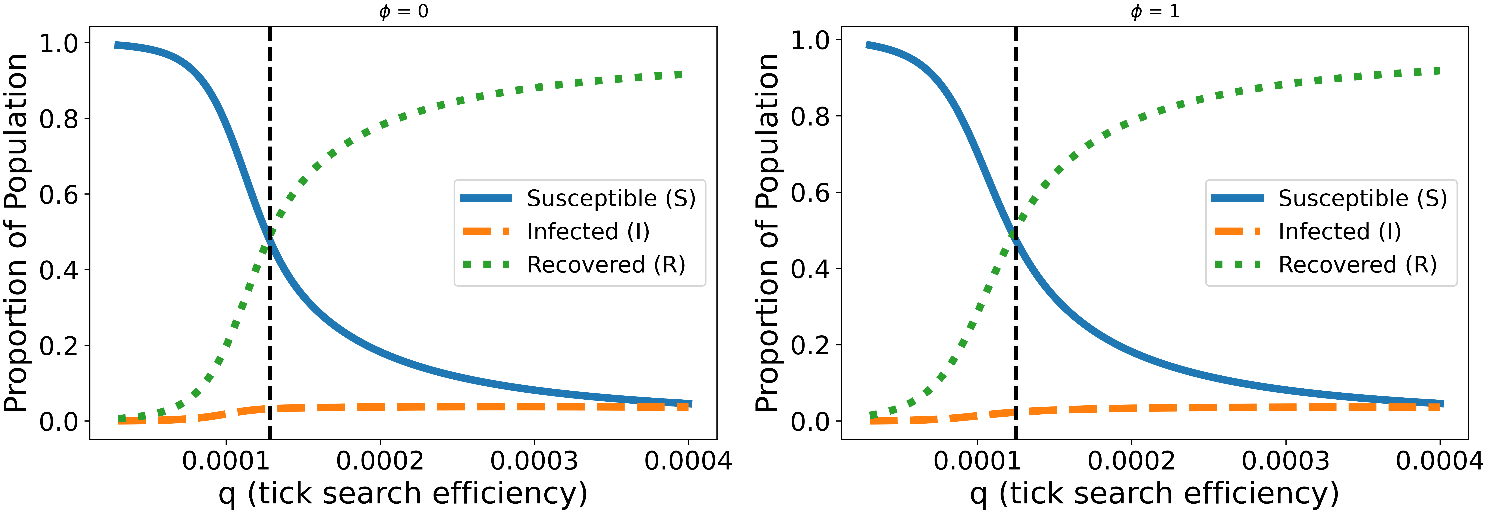


**Figure S2** – Varying the rate at which susceptible hosts produce immune offspring ($\emptyset$) from 0 (left panel) to 1 (right panel) has negligible impact on the relative equilibrium frequencies of susceptible ($S$), infected ($I$), and recovered ($R$) hosts at $t=1000$.
