# Supplementary material for: Tick ecology and host-finding efficiency interact to determine disease risk: a model of heartwater dynamics
Source: Parasitology. 2025 Jul 14;152(9):975–85. doi: 10.1017/S0031182025100553 (PMC12644940; doi:10.1017/S0031182025100553)
Supplement: Fisher and Vineer supplementary material 1 — Fisher and Vineer supplementary material [file S0031182025100553sup001.pdf]

```
In [37]: import numpy as np
import matplotlib.pyplot as plt
from matplotlib.ticker import MaxNLocator
from SALib.sample import saltelli
from SALib.analyze import sobol
from scipy.integrate import odeint
from mpl_toolkits.mplot3d import Axes3D
from matplotlib import cm
from scipy.stats import qmc
from sklearn.preprocessing import PolynomialFeatures
from sklearn.linear_model import LinearRegression

import warnings
warnings.filterwarnings('ignore')
```

Tick ecology and host-finding efficiency interact to determine disease risk: a model of heartwater dynamics

Authors: Adam Fisher & Hannah Rose Vineer

State variables:

Host Population

- S: Number of **susceptible hosts**.
- I: Number of **infected hosts**.
- R: Number of **recovered (immune) hosts**.
- $N = S + I + R$: **Total host population**.

Tick Population

- T_S: Number of **susceptible ticks**.
- T_I: Number of **infected ticks**.
- $T = T_S + T_I$: **Total tick population**.

Parameters:

Host Parameters

- β_{TH}: **Transmission rate** from ticks to hosts.
- γ: **Recovery rate** of infected hosts.
- δ: **Rate of immunity loss** in recovered hosts.
- μ_I: **Increase in death rate** for hosts due to heartwater infection.
- b_H: **Birth rate** for hosts.
- ϕ: **Rate of innate immunity** in calves born of susceptible hosts.
- K: **Carrying capacity** for hosts - cattle per km^2.
- q: **Tick search efficiency** (probability of successful search when N=1).

Tick Parameters

- β_{HT}: **Transmission rate** from hosts to ticks.
- μ_{T_I}: **Increase in death rate** for ticks due to heartwater pathogen.
- b_T: **Birth rate** for ticks.
- m: **Maximum number of ticks** per host in a saturated population.
- α: **Number of days** a tick can go without feeding before starvation.

```
In [40]: # Host parameters
beta_TH = 0.8      # Transmission rate from ticks to hosts
gamma = 0.05        # Recovery rate of hosts
delta = 0.005       # Rate at which recovered hosts lose immunity
mu_I = 0.067        # Increase in death rate for infected hosts
b_H = 0.02          # Intrinsic growth rate for hosts
K = 100          # Carrying capacity of hosts
q = 0.0004            # Tick search efficiency
phi = 0.5          #frequency of innate birth immunity

# Tick parameters
beta_HT = 0.12      # Transmission rate from hosts to ticks
mu_T_I = 0      # Increase in death rate for ticks carrying heartwater pathogen
b_T = 0.08          # Intrinsic growth rate for ticks
m = 50             # Maximum number of ticks per host
alpha = 518      #Number of days ticks can go without feeding before starving to death
```

Model Equations

Host-finding rate is derived from q (a discrete time probability), and N. The per tick probability of finding a host in 24 hours when $N > 1$ is $1-(1-q)^N$. The analogous host-finding rate is thus,

$$\lambda_q = -N\ln(1-q)$$

Host Equations

1. **Susceptible Hosts (S)**

$$\frac{dS}{dt} = b_H\left(1-\frac{N}{K}\right)S(1-\phi) + \frac{\delta}{1+\frac{T_I}{N}\lambda_q\beta_{TH}}R - S\frac{T_I}{N}\lambda_q\beta_{TH}$$

   - **Birth rate** (day^{-1}) into S: $b_H\left(1-\frac{N}{K}\right)S(1-\phi)$
   - **Rate of immunity loss** (day^{-1}) from R: $\frac{\delta}{1+\frac{T_I}{N}\lambda_q\beta_{TH}}$
   - **Infection rate** (day^{-1}): $S\frac{T_I}{N}\lambda_q\beta_{TH}$

2. **Infected Hosts (I)**

$$\frac{dI}{dt} = S\frac{T_I}{N}\lambda_q\beta_{TH} - \gamma I - \mu_I I$$

   - **Infection rate** (day^{-1}): $S\frac{T_I}{N}\lambda_q\beta_{TH}$
   - **Recovery rate** (day^{-1}): γI
   - **Death rate** (day^{-1}) due to heartwater: $\mu_I I$

3. **Recovered Hosts (R)**

$$\frac{dR}{dt} = b_H\left(1-\frac{N}{K}\right)(R+S\phi) + \gamma I - \frac{\delta}{1+\frac{T_I}{N}\lambda_q\beta_{TH}}R$$

   - **Birth rate** (day^{-1}) into R: $b_H\left(1-\frac{N}{K}\right)(R+S\phi)$
   - **Recovery rate of infecteds** (day^{-1}): γI
   - **Rate of immunity loss** (day^{-1}) from R: $\frac{\delta}{1+\frac{T_I}{N}\lambda_q\beta_{TH}}$

Tick Equations

4. **Susceptible Ticks (T_S)**

$$\frac{dT_S}{dt} = b_T\left(1-\frac{T}{mN}\right)T - \beta_{HT}T_S\frac{I}{N}\lambda_q - \frac{1}{\alpha(1+\lambda_q)}T_S$$

   - **Birth rate** (day^{-1}) of T_S: $b_T\left(1-\frac{T}{mN}\right)T$
   - **Infection rate** (day^{-1}): $\beta_{HT}T_S\frac{I}{N}\lambda_q$
   - **Death rate due to starvation** (day^{-1}): $\frac{1}{\alpha(1+\lambda_q)}T_S$

5. **Infected Ticks (T_I)**

$$\frac{dT_I}{dt} = \beta_{HT}T_S\frac{I}{N}\lambda_q - \mu_{T_I}T_I - \frac{1}{\alpha(1+\lambda_q)}T_I$$

   - **Infection rate** (day^{-1}): $\beta_{HT}T_S\frac{I}{N}\lambda_q$
   - **Death rate** (day^{-1}) due to carrying heartwater pathogen: $\mu_{T_I}T_I$
   - **Deaths due to starvation**: $\frac{1}{\alpha(1+\lambda_q)}T_I$

```
In [43]: # Initial conditions
S0 = 100          # Initial susceptible hosts
I0 = 0            # Initial infected hosts
R0 = 0             # Initial recovered hosts

T_S0 = 1000        # Initial susceptible ticks
T_I0 = 1         # Initial infected ticks

# Time vector
t = np.linspace(0, 1000, 10000)

# Differential equations
def deriv(y, t, beta_TH, beta_HT, gamma, delta, mu_I, b_H, b_T, K,
          mu_T_I, m, q, phi, alpha):
    S, I, R, T_S, T_I = y
    N = S + I + R
    T = T_S + T_I


    lambDa = -np.log(1-q)

    tick_bite_rate = lambDa * N
    tick_infection_rate = T_S * (I / N) * tick_bite_rate * beta_HT

    starvation_rate = 1/(alpha*(1+tick_bite_rate))

    host_infection_rate = S * (T_I / N) * tick_bite_rate * beta_TH

    #Repeated bite rate of recovered hosts
    recovered_bites = (T_I / N) * tick_bite_rate * beta_TH

    # Host equations
    dSdt = (b_H) * (1 - N / K) * S * (1 - phi) + delta/(1+recovered_bites) * R - host_infection_rate
    dIdt = host_infection_rate - gamma * I - mu_I * I
    dRdt = (b_H) * (1 - N / K) * (R + S * phi) + gamma * I - delta/(1+recovered_bites) * R

    # Tick capacity
    tick_capacity = m * N
    if tick_capacity <= 0:
        tick_capacity = 1e-6
    d_t = 0.0001
    # Tick birth rate
    tick_birth_rate = b_T * (1 - T / tick_capacity)

    # Tick equations
    dT_Sdt = tick_birth_rate * T - tick_infection_rate  - starvation_rate * T_S
    dT_Idt = tick_infection_rate - mu_T_I * T_I - starvation_rate * T_I

    return [dSdt, dIdt, dRdt, dT_Sdt, dT_Idt]
```

The equations can be integrated over time to visualise temporal population dynamics.

```
In [46]: # Initial condition vector
y0 = [S0, I0, R0, T_S0, T_I0]

# Integrate the differential equations over the time grid t
ret = odeint(deriv, y0, t, args=(beta_TH, beta_HT, gamma, delta, mu_I, b_H, b_T, K, mu_T_I, m, q, phi, alpha))
S, I, R, T_S, T_I = ret.T
```

```
In [48]: # Create side-by-side plots
fig, (ax1, ax2) = plt.subplots(1, 2, figsize=(14, 6))

# Host dynamics
ax1.plot(t, S, 'b', label='Susceptible Hosts', lw=4)
ax1.plot(t, I, 'r', label='Infected Hosts', lw=4)
ax1.plot(t, R, 'g', label='Recovered Hosts', lw=4)
ax1.set_xlabel('Time')
ax1.set_ylabel('Host Population')
ax1.legend()
ax1.grid(True)

# Tick dynamics
ax2.plot(t, T_S, 'c', label='Susceptible Ticks', lw=4)
ax2.plot(t, T_I, 'm', label='Infected Ticks', lw=4)
ax2.set_xlabel('Time')
ax2.set_ylabel('Tick Population')
ax2.legend()
ax2.grid(True)

plt.tight_layout()
plt.show()
```

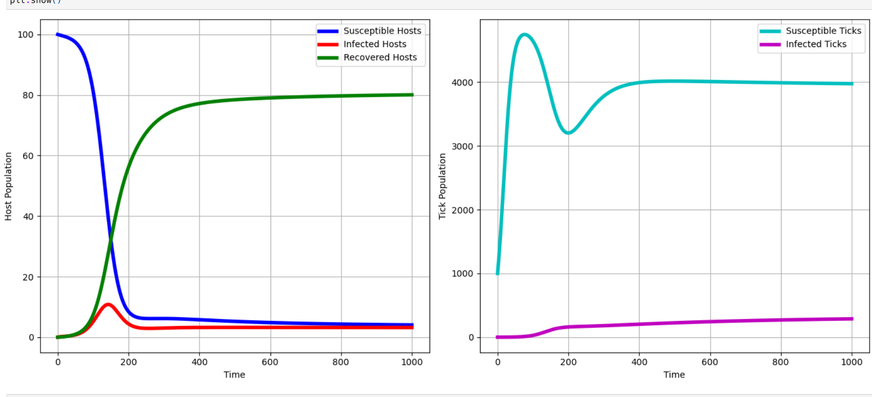


```
In [50]: print(I[-1]/(I[-1]+S[-1]+R[-1]))
print(T_I[-1]/(T_I[-1]+T_S[-1]))

0.03649621181988585
0.06761981916735839
```

Analyse long-term equilibria (t=1000)

```
In [71]: # Time and q values
t = np.linspace(0, 1000, 10000)  # Time points
q_values = np.linspace(0.00003, 0.0004, 200)  # Range of q values

# Initial conditions
S0, I0, R0 = 100, 0, 0  # Initial host population
T_S0, T_I0 = 1000, 10    # Initial tick population
y0 = [S0, I0, R0, T_S0, T_I0]

# K values for the 4 subplots
K_values = [50, 100, 150, 200]

# Prepare the figure
fig, axes = plt.subplots(2, 2, figsize=(14, 10))
axes = axes.flatten()

# Simulate for each K value
for i, K in enumerate(K_values):
    S_proportions, I_proportions, R_proportions = [], [], []

    for q in q_values:
        # Solve the differential equations
        result = odeint(deriv, y0, t, args=(beta_TH, beta_HT, gamma, delta, mu_I, b_H, b_T, K, mu_T_I, m, q, phi, alpha))
        S, I, R = result[-1, 0], result[-1, 1], result[-1, 2]  # Final values at t = 1000
        N = S + I + R  # Total population at equilibrium

        # Proportions of S, I, and R
        S_proportions.append(S / N)
        I_proportions.append(I / N)
        R_proportions.append(R / N)

    threshold = np.argmax(np.array(R_proportions) >= np.array(S_proportions))

    # Plot the results
    ax = axes[i]
    ax.plot(q_values, S_proportions, label='Susceptible (S)', linestyle='-', lw=5)
    ax.plot(q_values, I_proportions, label='Infected (I)', linestyle='--', lw=5)
    ax.plot(q_values, R_proportions, label='Recovered (R)', linestyle=':', lw=5)
    ax.axvline(x=q_values[threshold], color="black", linestyle='--', lw=2.5)
    ax.set_title(f"$K$ = {K}", fontsize=15)
    ax.set_xlabel("$q$", fontsize=20)
    ax.set_ylabel("Proportion at $t$=1000", fontsize=20)
    ax.tick_params(axis='both', which='both', labelsize=17)
    ax.legend(fontsize=15)
    ax.grid(False)

# Adjust layout and display
plt.tight_layout()

fig.savefig("figure4.tif",
            dpi=600,
            format="tiff",
            pil_kwargs={"compression": "tiff_lzw"},
            bbox_inches="tight", pad_inches=0.03)

plt.show()
```

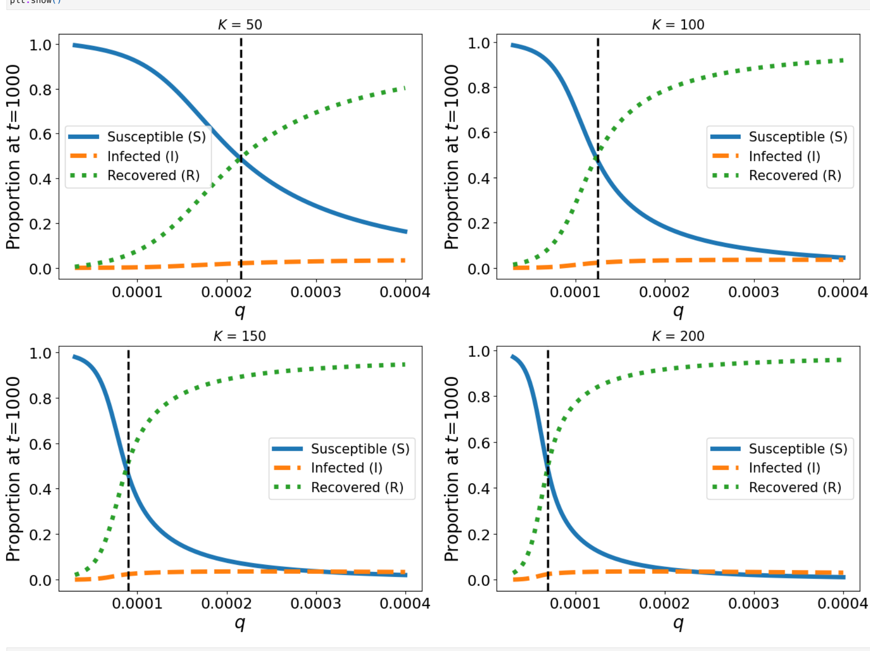


```
In [54]: # Parameter ranges
K_values = np.linspace(50, 200, 20)
m_values = np.linspace(25, 100, 20)

# Placeholder for threshold q values
threshold_q = np.full((len(K_values), len(m_values)), np.nan)

# Compute thresholds
for i, K in enumerate(K_values):
    for j, m in enumerate(m_values):
        found_threshold = False
        for q in q_values:
            result = odeint(deriv, y0, t, args=(beta_TH, beta_HT, gamma, delta, mu_I, b_H, b_T, K, mu_T_I, m, q, phi, alpha))
            S, I, R = result[-1, 0], result[-1, 1], result[-1, 2]  # Final values
            N = S + I + R  # Total population

            # Check if the threshold condition is met
            if R / N >= S / N:
                threshold_q[i, j] = q
                found_threshold = True
                break  # Exit the q loop early
        if not found_threshold:
            print(f"q threshold not found for parameters K={K}, m={m}")

# Create a 3D surface plot
K_mesh, m_mesh = np.meshgrid(K_values, m_values, indexing='ij')

fig = plt.figure(figsize=(10, 8))
ax = fig.add_subplot(111, projection='3d')
surf = ax.plot_surface(K_mesh, m_mesh, threshold_q, cmap='viridis', edgecolor='none')

#ax.view_init(elev=20, azim=5)

# Add labels and title
ax.set_xlabel('$K$', fontsize=14)
ax.set_ylabel('$m$', fontsize=14)
ax.set_zlabel('$q$ threshold', labelpad=20, fontsize=14)
ax.tick_params(axis='z', pad=10)

# Add color bar
fig.colorbar(surf, ax=ax, shrink=0.5, aspect=10, pad=0.1)

plt.tight_layout()

fig.savefig("figure5.tif",
            dpi=600,
            format="tiff",
            pil_kwargs={"compression": "tiff_lzw"},
            bbox_inches="tight", pad_inches=0.03)

plt.show()
```

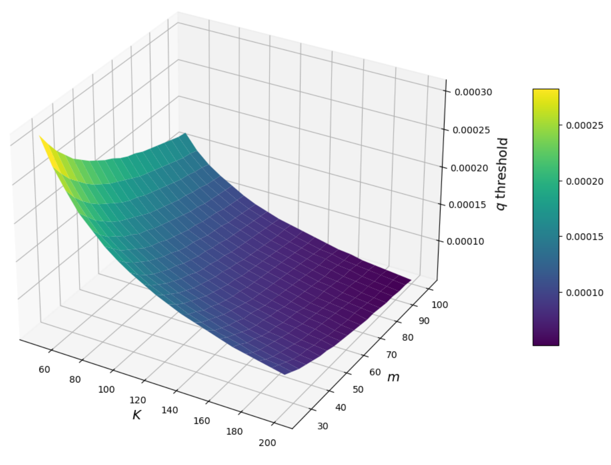


```
In [75]: # Define phi values for the 4 subplots
phi_values = [0, 1]

# Fix K for all plots
K = 100

#Fix m
m = 50

# Prepare the figure
fig, axes = plt.subplots(1, 2, figsize=(14, 5))
axes = axes.flatten()

# Simulate for each phi value
for i, phi in enumerate(phi_values):
    S_proportions, I_proportions, R_proportions = [], [], []

    for q in q_values:
        # Solve the differential equations
        result = odeint(deriv, y0, t, args=(beta_TH, beta_HT, gamma, delta, mu_I, b_H, b_T, K, mu_T_I, m, q, phi, alpha))
        S, I, R = result[-1, 0], result[-1, 1], result[-1, 2]  # Final values at t = 1000
        N = S + I + R  # Total population at equilibrium

        # Proportions of S, I, and R
        S_proportions.append(S / N)
        I_proportions.append(I / N)
        R_proportions.append(R / N)

    threshold = np.argmax(np.array(R_proportions) >= np.array(S_proportions))

    # Plot the results
    ax = axes[i]
    ax.plot(q_values, S_proportions, label='Susceptible (S)', linestyle='-', lw=5)
    ax.plot(q_values, I_proportions, label='Infected (I)', linestyle='--', lw=5)
    ax.plot(q_values, R_proportions, label='Recovered (R)', linestyle=':', lw=5)
    ax.axvline(x=q_values[threshold], color="black", linestyle='--', lw=2.5)
    ax.set_title(f"$\phi$ = {phi}")
    ax.set_xlabel("q (tick search efficiency)", fontsize=20)
    ax.set_ylabel("Proportion of Population", fontsize=20)
    ax.tick_params(axis='both', which='both', labelsize=17)
    ax.legend(fontsize=15)
    ax.grid(False)

# Adjust layout and display
plt.tight_layout()

fig.savefig("figureS2.tif",
            dpi=600,
            format="tiff",
            pil_kwargs={"compression": "tiff_lzw"},
            bbox_inches="tight", pad_inches=0.03)

plt.show()
```

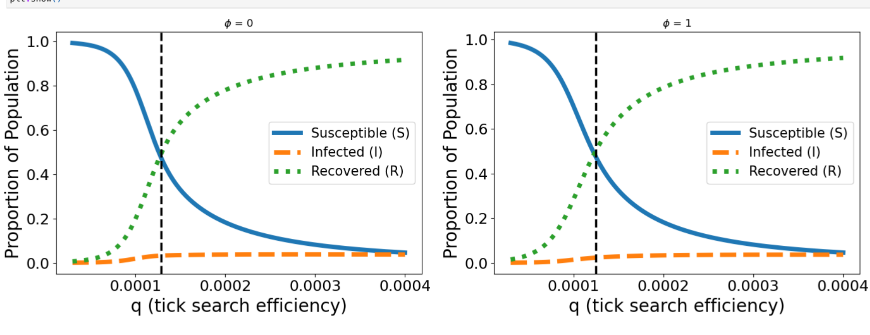

Derivation of the Basic Reproduction Number (R_0) Using the Next-Generation Matrix

1. Identifying Infected Compartments

For the host-tick disease dynamics model, the infected compartments are:

- **Infected Hosts** (I)
- **Infected Ticks** (T_I)

2. Defining New Infection Terms (F) and Transition Terms (V)

New Infections (F)

- **Infected Hosts** (I):

$$F_I = \beta_{TH}S\frac{T_I}{N}\lambda_q$$

- **Infected Ticks** (T_I):

$$F_{T_I} = \beta_{HT}T_S\frac{I}{N}\lambda_q$$

Transition Terms/rate at which individuals leave the infected class (V)

- **Infected Hosts** (I):

$$V_I = (\gamma+\mu_I)I$$

- **Infected Ticks** (T_I):

$$V_{T_I} = \left(\mu_{T_I} + \frac{1}{\alpha(1+\alpha)}\right)T_I$$

3. Constructing the Next-Generation Matrix (K)

Step 1.1: Partial Derivatives (F)

$$\frac{\partial F_I}{\partial I} = 0$$

$$\frac{\partial F_I}{\partial T_I} = \beta_{TH}\lambda_q$$

(above, $\frac{S}{N}$ cancels out because we assume a naive population; thus, $S = N$)

$$\frac{\partial F_{T_I}}{\partial T_I} = 0$$

$$\frac{\partial F_{T_I}}{\partial I} = \beta_{HT}\frac{T_S}{N}\lambda_q$$

- **Matrix** (F):

$$F = \begin{bmatrix} 0 & \beta_{TH}\lambda_q \\ \beta_{HT}\frac{T_S}{N}\lambda_q & 0 \end{bmatrix}$$

Step 1.2: Partial Derivatives (V)

$$\frac{\partial V_I}{\partial I} = \gamma + \mu_I$$

$$\frac{\partial V_I}{\partial T_I} = 0$$

$$\frac{\partial V_{T_I}}{\partial T_I} = \mu_{T_I} + \frac{1}{\alpha(1+\alpha)}$$

$$\frac{\partial V_{T_I}}{\partial I} = 0$$

- **Matrix** (V):

$$V = \begin{bmatrix} \gamma+\mu_I & 0 \\ 0 & \mu_{T_I} + \frac{1}{\alpha(1+\alpha)} \end{bmatrix}$$

Step 2: Inverse of Matrix (V)

$$V^{-1} = \begin{bmatrix} \frac{1}{\gamma+\mu_I} & 0 \\ 0 & \frac{1}{\mu_{T_I}+\frac{1}{\alpha(1+\alpha)}} \end{bmatrix}$$

Step 3: Next-Generation Matrix ($K = FV^{-1}$)

$$K = FV^{-1} = \begin{bmatrix} 0 & \frac{\beta_{TH}\lambda_q}{\gamma+\mu_I} \\ \frac{\beta_{HT}\frac{T_S}{N}\lambda_q}{\mu_{T_I}+\frac{1}{\alpha(1+\alpha)}} & 0 \end{bmatrix}$$

4. Calculating the Basic Reproduction Number (R_0)

The basic reproduction number (R_0) is the **spectral radius** (largest eigenvalue) of the next-generation matrix K.

For a 2×2 matrix of the form:

$$K = \begin{bmatrix} 0 & a \\ b & 0 \end{bmatrix}$$

The eigenvalues are $\lambda = \pm\sqrt{a \cdot b}$. Therefore, the spectral radius is:

$$R_0 = \sqrt{a \cdot b}$$

Applying this to our matrix (K):

$$R_0 = \sqrt{\frac{\beta_{TH}\cdot\beta_{HT}\cdot\frac{T_S}{N}\cdot\lambda_q^2}{(\gamma+\mu_I)\cdot\left(\mu_{T_I}+\frac{1}{\alpha(1+\alpha)}\right)}}$$

5. Condition for Disease Spread

The disease can invade and persist in the population if:

$$R_0 > 1$$

Substituting the expression for R_0:

$$\sqrt{\frac{\beta_{TH}\cdot\beta_{HT}\cdot\frac{T_S}{N}\cdot\lambda_q^2}{(\gamma+\mu_I)\cdot\left(\mu_{T_I}+\frac{1}{\alpha(1+\alpha)}\right)}} > 1$$

Squaring both sides to eliminate the square root:

$$\frac{\beta_{TH}\cdot\beta_{HT}\cdot\frac{T_S}{N}\cdot\lambda_q^2}{(\gamma+\mu_I)\cdot\left(\mu_{T_I}+\frac{1}{\alpha(1+\alpha)}\right)} > 1$$

Which simplifies to:

$$\beta_{TH}\cdot\beta_{HT}\cdot\frac{T_S}{N}\cdot\lambda_q^2 > (\gamma+\mu_I)\cdot\left(\mu_{T_I}+\frac{1}{\alpha(1+\alpha)}\right)$$

This analytical expression for R_0 provides a clear threshold to assess whether interventions are needed to control the disease outbreak.

Next step: sensitivity analysis of R_0

Let's start with Latin Hypercube Sampling to get a qualitative visual overview of the magnitude and direction of the sensitivity R_0 to the relevant parameters.

```
In [57]: def compute_R0(beta_TH, beta_HT, q, gamma, mu_I, mu_T_I, N, T_S, alpha):
    """
    Compute the basic reproduction number R0.

    Parameters:
    - beta_TH: Transmission rate from ticks to hosts
    - beta_HT: Transmission rate from hosts to ticks
    - q: Tick search efficiency
    - gamma: Recovery rate of infected hosts
    - mu_I: Death rate for infected hosts
    - mu_T_I: Death rate for infected ticks
    - N: Total host population
    - T_S: Some parameter T_S
    - alpha: Parameter alpha

    Returns:
    - R0: Basic reproduction number
    """
    # Calculate the instantaneous rate lambda for one host
    lambda_rate = -np.log(1 - q)
    tick_bite_rate = N * lambda_rate
    starvation_rate =  1/(alpha*(1+tick_bite_rate))

    # The continuous-time probability that an event occurs is:
    # 1 - e^(-N * lambda_rate)
    # and the probability that no event occurs is e^(-N * lambda_rate)

    # Compute R0 using the instantaneous rate formulation
    # Adjust the expression to use np.log and np.exp for array operations
    R0 = np.sqrt((beta_TH * beta_HT * (T_S / N) * tick_bite_rate**2) /
                 ((gamma + mu_I) * (mu_T_I + starvation_rate)))
    return R0


# Define parameter ranges for LHS
parameters = [
    {'name': 'beta_TH', 'label': r'$\beta_{TH}$', 'range': (0.7, 0.9)},
    {'name': 'beta_HT', 'label': r'$\beta_{HT}$', 'range': (0.05, 0.32)},
    {'name': 'q', 'label': r'$q$', 'range': (0.00003, 0.0004)},
    {'name': 'gamma', 'label': r'$\gamma$', 'range': (0.01, 0.1)},
    {'name': 'mu_I', 'label': r'$\mu_I$', 'range': (0.03, 0.1)},
    {'name': 'mu_T_I', 'label': r'$\mu_{T_I}$', 'range': (0, 0.05)},
    {'name': 'N', 'label': r'$N$', 'range': (50, 200)},
    {'name': 'T_S', 'label': r'$T_S$', 'range': (1250, 20000)},
    {'name': 'alpha', 'label': r'$\alpha$', 'range': (300, 600)}
]

#N=K  #assume hosts are at carrying capacity

# Number of samples
num_samples = 2048

# Initialize Latin Hypercube Sampler
sampler = qmc.LatinHypercube(d=9)
sample = sampler.random(n=num_samples)

# Scale the samples to the parameter ranges
lower_bounds = [param['range'][0] for param in parameters]
upper_bounds = [param['range'][1] for param in parameters]
sample_scaled = qmc.scale(sample, lower_bounds, upper_bounds)

# Extract individual parameter arrays
beta_TH_samples = sample_scaled[:, 0]
beta_HT_samples = sample_scaled[:, 1]
q_samples = sample_scaled[:, 2]
gamma_samples = sample_scaled[:, 3]
mu_I_samples = sample_scaled[:, 4]
mu_T_I_samples = sample_scaled[:, 5]
N_samples = sample_scaled[:, 6]
T_S_samples = sample_scaled[:, 7]
alpha_samples = sample_scaled[:, 8]

# Compute R0 for each sample
R0_samples = compute_R0(beta_TH_samples, beta_HT_samples, q_samples, gamma_samples, mu_I_samples, mu_T_I_samples, N_samples, T_S_samples, alpha_samples)

# Create a 3x3 grid of subplots
fig, axs = plt.subplots(3, 3, figsize=(20, 20))
fig.subplots_adjust(hspace=0.4, wspace=0.3)


# Create custom legend elements
scatter_proxy = plt.Line2D([0], [0], marker='o', color='w', label='Samples',
                           markerfacecolor='gray', markersize=10, alpha=0.6, markeredgecolor='w')
line_proxy = plt.Line2D([0], [0], color='red', lw=2, label='Polynomial Trendline')
threshold_proxy = plt.Line2D([0], [0], color='blue', lw=2, linestyle='--', label=r'$R_0=1$ Threshold')

# Add legend to the first panel
#ax0.legend(handles=[scatter_proxy, line_proxy, threshold_proxy], loc='center',
#          # fontsize=14, frameon=True)

# Define subplot positions and corresponding parameters
subplot_params = [
    (0, 0, 'beta_TH'),
    (0, 1, 'beta_HT'),
    (0, 2, 'q'),
    (1, 0, 'gamma'),
    (1, 1, 'mu_I'),
    (1, 2, 'mu_T_I'),
    (2, 0, 'N'),
    (2, 1, 'T_S'),
    (2, 2, 'alpha')
]

# Helper function to plot sensitivity
def plot_sensitivity(ax, x, y, xlabel, title):
    """
```

```python
    Plot R0 vs a single parameter with a polynomial trend line.

    Parameters:
    - ax: Matplotlib Axes object
    - x: Independent variable array
    - y: Dependent variable array (R0)
    - xlabel: Label for the x-axis
    - title: Title of the plot
    """
    # Scatter plot of sampled data
    scatter = ax.scatter(x, y, alpha=0.6, edgecolors='w', s=50, label='Samples', color='gray')

    # Fit a 3rd degree polynomial
    poly = PolynomialFeatures(degree=3)
    x_poly = poly.fit_transform(x.reshape(-1, 1))
    model = LinearRegression()
    model.fit(x_poly, y)
    y_poly_pred = model.predict(x_poly)

    # Sort the values for a smooth curve
    sorted_indices = np.argsort(x)
    x_sorted = x[sorted_indices]
    y_sorted = y_poly_pred[sorted_indices]

    # Plot the polynomial trend line
    ax.plot(x_sorted, y_sorted, color='red', linewidth=4, label='Polynomial Trendline')

    # Plot the R0=1 threshold line
    ax.axhline(1, color='blue', linestyle='--', linewidth=3, label=r'$R_0=1$ Threshold')

    ax.set_ylim(0, 10)

    # Set axis labels and title
    ax.set_xlabel(xlabel, fontsize=30)
    ax.set_ylabel(r'$R_0$', fontsize=30)
    ax.set_title(title, fontsize=16)
    ax.tick_params(axis='both', which='both', labelsize=17)
    ax.locator_params(axis="x", nbins=6)


    # Add grid
    ax.grid(True, linestyle='--', alpha=0.6)

# Iterate over each subplot and plot the corresponding parameter
for row, col, param_name in subplot_params:
    # Find the parameter dictionary
    param = next(item for item in parameters if item['name'] == param_name)
    label = param['label']

    # Select data based on parameter
    if param_name == 'beta_TH':
        x = beta_TH_samples
    elif param_name == 'beta_HT':
        x = beta_HT_samples
    elif param_name == 'q':
        x = q_samples
    elif param_name == 'gamma':
        x = gamma_samples
    elif param_name == 'mu_I':
        x = mu_I_samples
    elif param_name == 'mu_T_I':
        x = mu_T_I_samples
    elif param_name == 'N':
        x = N_samples
    elif param_name == 'T_S':
        x = T_S_samples
    elif param_name == 'alpha':
        x = alpha_samples
    else:
        raise ValueError("Unknown parameter name.")

    y = R0_samples
    xlabel = label
    title ="" # f'$R_0$ vs {label}'

    # Plot on the corresponding subplot
    ax = axs[row, col]
    plot_sensitivity(ax, x, y, xlabel, title)

plt.tight_layout()

fig.savefig("figure1.tif",
            dpi=600,
            format="tiff",
            pil_kwargs={"compression": "tiff_lzw"},
            bbox_inches="tight", pad_inches=0.08)

plt.show()
```

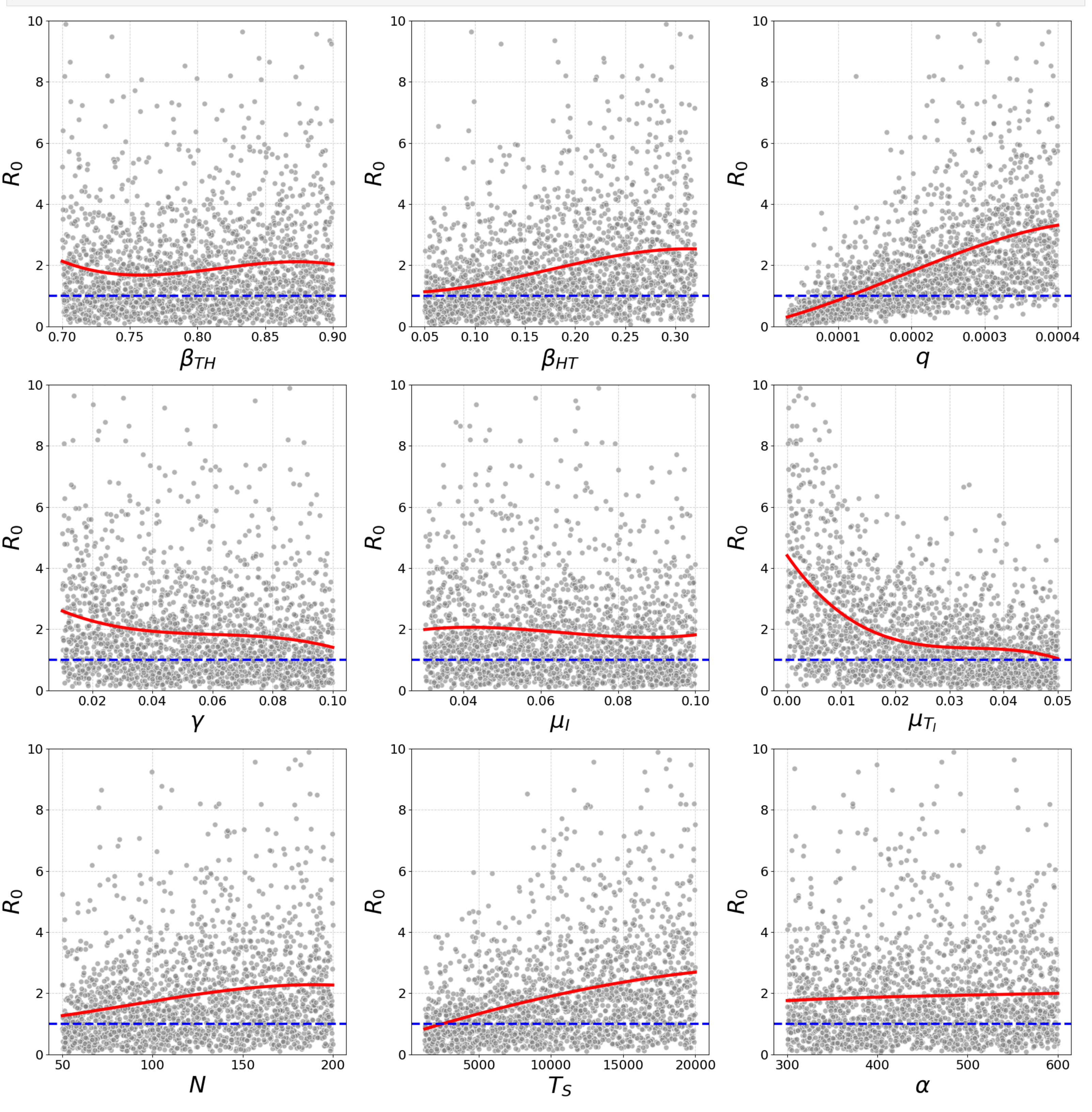

Next step: formal quantification of parameter sensitivity

Now, it would be good to formally quantify the effect size of each of these parameters. Using **Sobol's Sensitiviy Index** we can produce estimates of the impact of variation in each parameter in isolation AND their total impact (impact in isolation + impact of interactions).

In [60]:
```python
# Define the problem for Sobol analysis
param_names = [param['name'] for param in parameters]
param_bounds = [param['range'] for param in parameters]

problem = {
    'num_vars': len(param_names),
    'names': param_names,
    'bounds': param_bounds
}

# Generate samples using Saltelli's method
saltelli_samples = saltelli.sample(problem, num_samples, calc_second_order=True)

# Evaluate R0 for each sample
def evaluate_R0(samples):
    results = []
    for sample in samples:
        R0_value = compute_R0(*sample)
        results.append(R0_value)
    return np.array(results)

R0_sobol_samples = evaluate_R0(saltelli_samples)

# Perform Sobol analysis
sobol_results = sobol.analyze(problem, R0_sobol_samples, print_to_console=False)

# Extract Sobol indices
first_order = sobol_results['S1']
total_effects = sobol_results['ST']
first_order_conf = sobol_results['S1_conf']
total_effects_conf = sobol_results['ST_conf']

# Mapping from parameter prefixes to LaTeX Greek symbols
greek_letters = {
    'beta': r'\beta',
    'mu': r'\mu',
    'gamma': r'\gamma',
    'delta': r'\delta',
    'alpha': r'\alpha',
    'theta': r'\theta'
}

# Create formatted labels using the mapping
formatted_labels = [
    f'${greek_letters.get(name.split("_")[0], name.split("_")[0])}_{{{"_".join(name.split("_")[1:])}}}$'
    if '_' in name else f'${greek_letters.get(name, name)}$'
    for name in param_names
]

# Plot Sobol indices
x_indices = np.arange(len(param_names))
bar_width = 0.4

fig, ax = plt.subplots(figsize=(12, 6))

plt.ylim(0, 0.6)  # Sets the minimum y-axis value to 0, and keeps the max automatic

# Add grid for better readability
ax.yaxis.grid(True, linestyle='--', alpha=0.7)

# First order indices
ax.bar(x_indices - bar_width/2, first_order, bar_width, yerr=first_order_conf, capsize=5, label='First Order',
       color='teal', edgecolor='black')

# Total order indices
ax.bar(x_indices + bar_width/2, total_effects, bar_width, yerr=total_effects_conf, capsize=5, label='Total Effect',
       color='coral', edgecolor='black')

# Customize plot
ax.set_xticks(x_indices)
ax.set_xticklabels(formatted_labels, rotation=45, ha='right', fontsize=15)
ax.set_ylabel('Sobol Index', fontsize=20)
ax.legend(fontsize=15)
plt.tight_layout()

fig.savefig("figure2.tif",
            dpi=600,
            format="tiff",
            pil_kwargs={"compression": "tiff_lzw"},
            bbox_inches="tight", pad_inches=0.08)

plt.show()
```

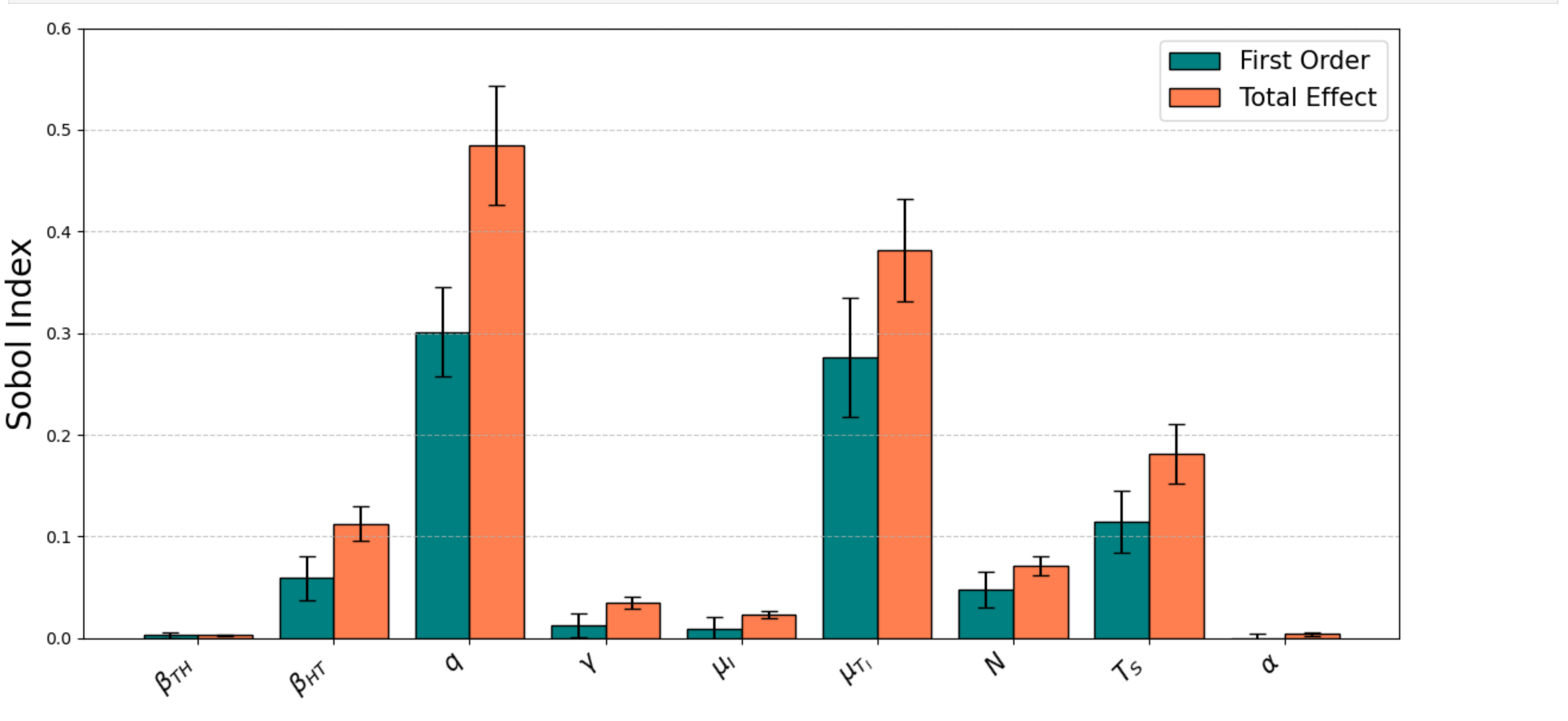

R_0 is most sensitive to parameters q, followed by μ_{T_I}, T_S, β_{HT}, and N. Moreover, the total effects ofthese parameters are significantly greater than their first order effects, suggesting important interactions are present......

Next step: investigate interactions between q and other parameters

To do this, we will generate a 3D surface plot. First we will look at interactions between the two most influential parameters (q and μ_{T_I})

In [62]:
```python
# Define a grid for q and mu_T_I
q_range = np.linspace(0.00003, 0.0004, 50)
mu_T_I_range = np.linspace(0, 0.05, 50)
q_grid, mu_T_I_grid = np.meshgrid(q_range, mu_T_I_range)

# Compute R0 values for the grid
R0_grid = compute_R0(
    beta_TH=0.8,  # Fixed value
    beta_HT=0.12,   # Fixed value
    q=q_grid,
    N=100,
    gamma=0.05,    # Fixed value
    mu_I=0.067,     # Fixed value
    mu_T_I=mu_T_I_grid,     # Fixed value
    T_S=2500,
    alpha=518      # Fixed value
)

# Create the 3D plot
fig = plt.figure(figsize=(10, 8))
ax = fig.add_subplot(111, projection='3d')

# Plot the surface
surf = ax.plot_surface(q_grid, mu_T_I_grid, R0_grid, cmap='viridis', edgecolor='none')

ax.view_init(elev=20, azim=130)

# Add labels and title
ax.set_xlabel(r'$q$', fontsize=18, labelpad=10)
ax.set_ylabel(r'$\mu_{T_I}$', fontsize=18, labelpad=10)
ax.set_zlabel(r'$R_0$', fontsize=18, rotation=90)
#ax.zaxis.label.set_rotation(90)
#ax.set_title('3D Surface Plot of $R_0$ vs $q$ and $T_S$', fontsize=14)

# Add a color bar
fig.colorbar(surf, ax=ax, shrink=0.5, aspect=10)

plt.tight_layout()

fig.savefig("figure3.tif",
            dpi=600,
            format="tiff",
            pil_kwargs={"compression": "tiff_lzw"},
            bbox_inches="tight", pad_inches=0.08)

plt.show()
```

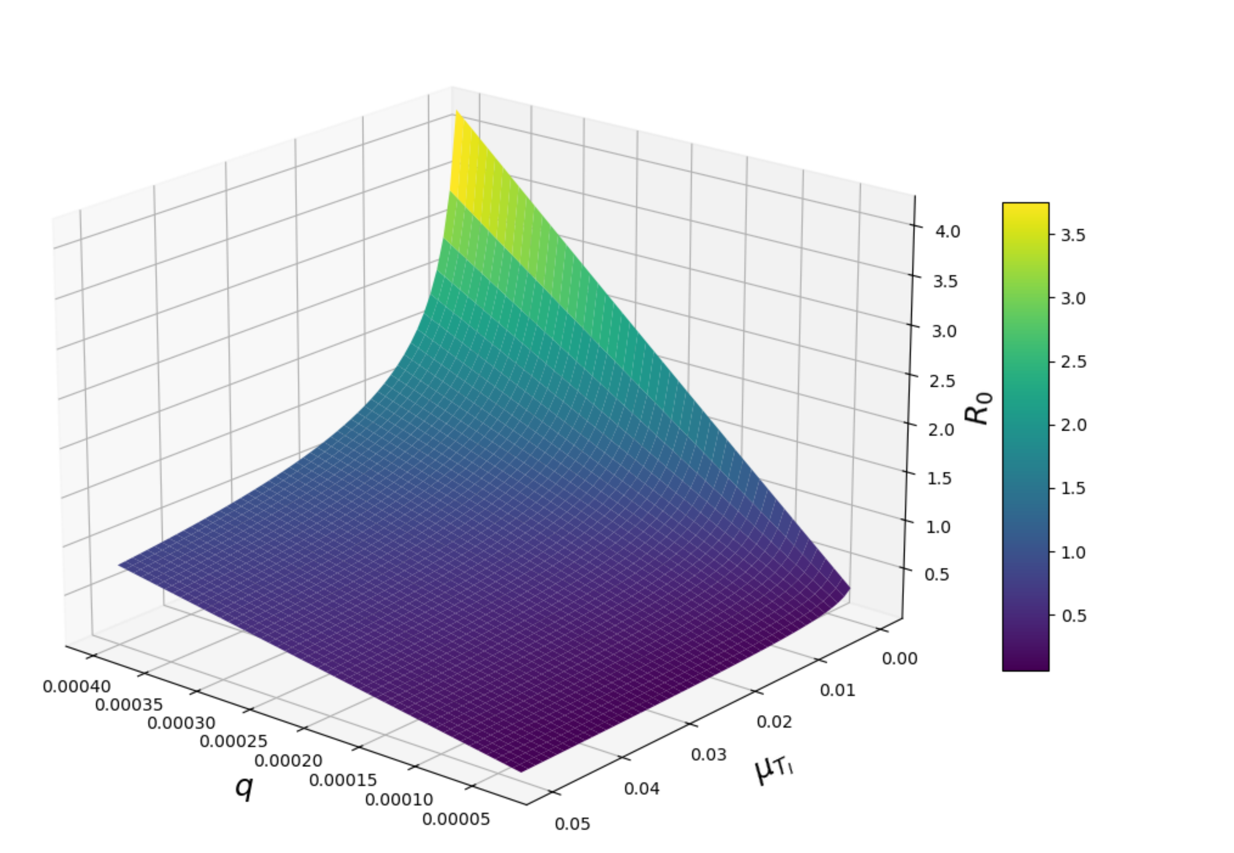

Even a small increase in tick mortality caused by E. ruminantium in can drastically reduce R_0, this effect is most obvious in the upper ranges of q.

In [64]:
```python
# Create a figure with 1 row and 3 columns of subplots (all 3D)
fig = plt.figure(figsize=(25, 7))

# Manually adjust the subplot parameters to reduce white space
#fig.subplots_adjust(left=0.05, right=1.2, top=0.95, bottom=0.05, wspace=0.1)

##################################
# Plot 1: R0 vs q and T_S        #
##################################
ax1 = fig.add_subplot(131, projection='3d')

# Define the grid for q and T_S
q_range = np.linspace(0.00003, 0.0004, 50)
T_S_range = np.linspace(1250, 5000, 50)
q_grid, T_S_grid = np.meshgrid(q_range, T_S_range)

# Compute R0 values (compute_R0() is assumed to be defined earlier)
R0_grid = compute_R0(
    beta_TH=0.8,
    beta_HT=0.12,
    q=q_grid,
    N=100,
    gamma=0.05,
    mu_I=0.067,
    mu_T_I=0,
    T_S=T_S_grid,
    alpha=518
)

# Plot the surface
surf1 = ax1.plot_surface(q_grid, T_S_grid, R0_grid, cmap='viridis', edgecolor='none')

# Set labels; rotate z-axis label to horizontal
ax1.set_xlabel(r'$q$', fontsize=14, labelpad=8)
ax1.set_ylabel(r'$T_S$', fontsize=14, labelpad=8)
#ax1.set_zlabel(r'$R_0$', fontsize=14, labelpad=8)
ax1.zaxis.label.set_rotation(0)
ax1.view_init(elev=20, azim=130)

# Add a color bar
fig.colorbar(surf1, ax=ax1, shrink=0.5, aspect=10, pad=0.12)

# Limit tick labels
ax1.xaxis.set_major_locator(MaxNLocator(5))
ax1.yaxis.set_major_locator(MaxNLocator(5))
ax1.zaxis.set_major_locator(MaxNLocator(5))


#########################################
# Plot 3: R0 vs q and β_HT              #
#########################################
ax2 = fig.add_subplot(132, projection='3d')

# Define the grid for q and β_HT
q_range = np.linspace(0.00003, 0.0004, 50)
beta_HT_range = np.linspace(0.05, 0.32, 50)
q_grid, beta_HT_grid = np.meshgrid(q_range, beta_HT_range)

# Compute R0 values
R0_grid = compute_R0(
    beta_TH=0.8,
    beta_HT=beta_HT_grid,
    q=q_grid,
    N=100,
    gamma=0.05,
    mu_I=0.067,
    mu_T_I=0,
    T_S=2500,
    alpha=518
)

# Plot the surface
surf3 = ax2.plot_surface(q_grid, beta_HT_grid, R0_grid, cmap='viridis', edgecolor='none')

# Set labels; rotate z-axis label to horizontal
ax2.set_xlabel(r'$q$', fontsize=14, labelpad=8)
ax2.set_ylabel(r'$\beta_{HT}$', fontsize=14, labelpad=8)
#ax2.set_zlabel(r'$R_0$', fontsize=14, labelpad=8)
ax2.zaxis.label.set_rotation(0)
ax2.view_init(elev=20, azim=130)

# Add a color bar
fig.colorbar(surf3, ax=ax2, shrink=0.5, aspect=10, pad=0.12)

# Limit tick labels
ax2.xaxis.set_major_locator(MaxNLocator(5))
ax2.yaxis.set_major_locator(MaxNLocator(5))
ax2.zaxis.set_major_locator(MaxNLocator(5))


##################################
# Plot 4: R0 vs q and N          #
##################################
ax3 = fig.add_subplot(133, projection='3d')

# Define the grid for q and N
q_range = np.linspace(0.00003, 0.0004, 50)
N_range = np.linspace(50, 200, 50)
q_grid, N_grid = np.meshgrid(q_range, N_range)

# Compute R0 values
R0_grid = compute_R0(
    beta_TH=0.8,
    beta_HT=0.12,
    q=q_grid,
    N=N_grid,
    gamma=0.05,
    mu_I=0.067,
    mu_T_I=0,
    T_S=2500,
    alpha=518
)

# Plot the surface
surf4 = ax3.plot_surface(q_grid, N_grid, R0_grid, cmap='viridis', edgecolor='none')

# Set labels; rotate z-axis label to horizontal
ax3.set_xlabel(r'$q$', fontsize=14, labelpad=8)
ax3.set_ylabel(r'$N$', fontsize=14, labelpad=8)
#ax3.set_zlabel(r'$R_0$', fontsize=14, labelpad=8)
ax3.zaxis.label.set_rotation(0)
ax3.view_init(elev=20, azim=130)

# Add a color bar
fig.colorbar(surf4, ax=ax3, shrink=0.5, aspect=10, pad=0.12)

# Limit tick labels
ax3.xaxis.set_major_locator(MaxNLocator(5))
ax3.yaxis.set_major_locator(MaxNLocator(5))
ax3.zaxis.set_major_locator(MaxNLocator(5))

fig.savefig("figureS1.tif",
            dpi=600,
            format="tiff",
            pil_kwargs={"compression": "tiff_lzw"},
            bbox_inches="tight", pad_inches=0.08)

plt.show()
```

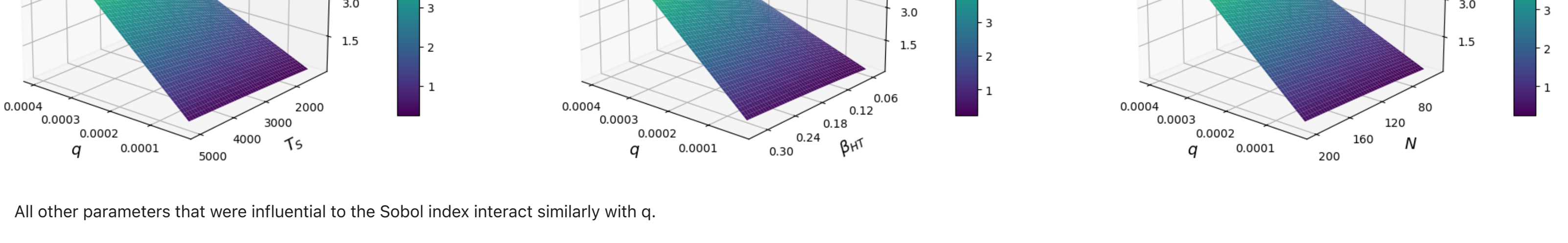

All other parameters that were influential to the Sobol index interact similarly with q.
